# A coronary artery disease-associated tRNA^Thr^ mutation altered mitochondrial function, apoptosis and angiogenesis

**DOI:** 10.1093/nar/gky1241

**Published:** 2018-12-12

**Authors:** Zidong Jia, Ye Zhang, Qiang Li, Zhenzhen Ye, Yuqi Liu, Changzhu Fu, Xiaohui Cang, Meng Wang, Min-Xin Guan

**Affiliations:** 1 Division of Medical Genetics and Genomics, The Children's Hospital, Zhejiang University School of Medicine, Hangzhou, Zhejiang 310058, China; 2 Institute of Genetics, Zhejiang University and Department of Human Genetics, Zhejiang University School of Medicine, Hangzhou, Zhejiang 310058, China; 3 Cardiac Department, PLA General Hospital, Beijing 100853, China; 4 Key lab of Reproductive Genetics, Ministry of Education of PRC, Zhejiang University, Hangzhou, Zhejiang 310058, China; 5 Joint Institute of Genetics and Genome Medicine between Zhejiang University and University of Toronto, Hangzhou, Zhejiang 310058, China

## Abstract

The tissue specificity of mitochondrial tRNA mutations remains largely elusive. In this study, we demonstrated the deleterious effects of tRNA^Thr^ 15927G>A mutation that contributed to pathogenesis of coronary artery disease. The m.15927G>A mutation abolished the highly conserved base-pairing (28C-42G) of anticodon stem of tRNA^Thr^. Using molecular dynamics simulations, we showed that the m.15927G>A mutation caused unstable tRNA^Thr^ structure, supported by decreased melting temperature and slower electrophoretic mobility of mutated tRNA. Using cybrids constructed by transferring mitochondria from a Chinese family carrying the m.15927G>A mutation and a control into mitochondrial DNA (mtDNA)-less human umbilical vein endothelial cells, we demonstrated that the m.15927G>A mutation caused significantly decreased efficiency in aminoacylation and steady-state levels of tRNA^Thr^. The aberrant tRNA^Thr^ metabolism yielded variable decreases in mtDNA-encoded polypeptides, respiratory deficiency, diminished membrane potential and increased the production of reactive oxygen species. The m.15927G>A mutation promoted the apoptosis, evidenced by elevated release of cytochrome *c* into cytosol and increased levels of apoptosis-activated proteins: caspases 3, 7, 9 and PARP. Moreover, the lower wound healing cells and perturbed tube formation were observed in mutant cybrids, indicating altered angiogenesis. Our findings provide new insights into the pathophysiology of coronary artery disease, which is manifested by tRNA^Thr^ mutation-induced alterations.

## INTRODUCTION

Cardiovascular diseases are the leading cause of death globally, including coronary artery disease (CAD), stroke, heart failure, hypertensive heart disease and cardiomyopathy (1–3). Of these, CAD typically occurs when part of the smooth, elastic lining inside a coronary artery (the arteries that supply blood to the heart muscle) develops atherosclerosis (4). However, the etiology of coronary artery disease is not well understood because of multi-factorial causes including environmental and inherited risk factors (5,6). Mitochondrial dysfunction plays an important role in cardiovascular pathophysiology, especially for myocardial infarction, cardiac hypertrophy, and heart failure (7–9). These mitochondrial causes of cell dysfunction include the disturbed mitochondrial energy metabolism, excessive generation of reactive oxygen species, changed Ca^2+^ concentrations and signaling, mitochondrial uncoupling and enhanced apoptosis (10,11). Human mtDNA encodes 13 subunits of oxidative phosphorylation system (OXPHOS), 2 rRNAs and 22 tRNAs required for translation (12). Mitochondrial tRNAs are the hot spots for mutations associated with cardiovascular diseases (13–17). The tRNA^Ile^ 4291T>C mutation has been associated with a cluster of metabolic defects, including hypertension, hypercholesterolemia and hypomagnesemia (18). These hypertension-associated tRNA mutations included the tRNA^Ile^ 4263A>G and 4295A>G, tRNA^Met^ 4435A>G, tRNA^Ala^ 5655A>G, tRNA^Leu(UUR)^ 3253T>C mutations and 4401A>G mutation in the junction of tRNA^Met^ and tRNA^Gln^ genes (15,19–26). Most recently, the tRNA^Thr^ 15927G>A was identified as the first mtDNA mutation associated with CAD (17,27). These tRNA mutations led to structural and functional consequences of tRNAs, including the processing of RNA precursors, stability, nucleotide modification and aminoacylation of tRNAs (20–25). However, the pathophysiology underlying these tRNA mutations, specifically the tissue specific effect, remains poorly understood.

**Figure 1.**
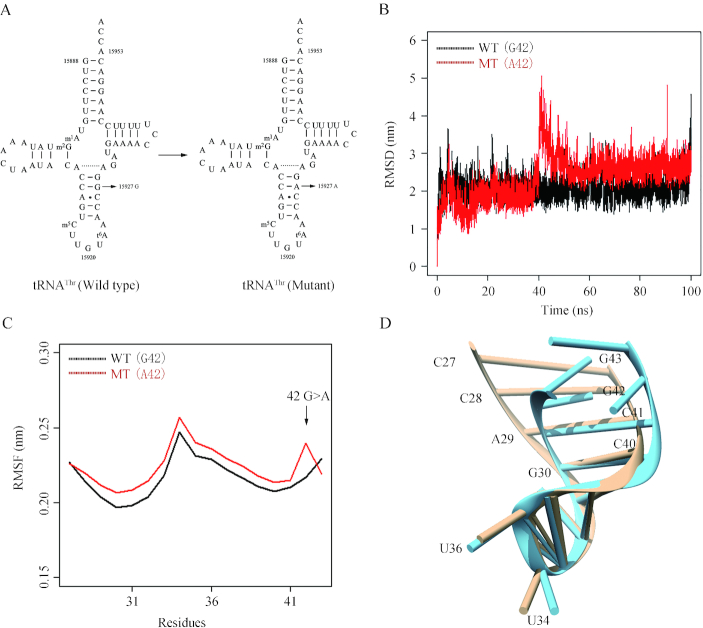
MD simulations on the anticodon stem-loop of wild-type and mutated tRNA^Thr^. (**A**) Cloverleaf structure of human mitochondrial tRNA^Thr^ (27–29). An arrow indicated the location of the m.15927 G>A mutation. (**B**) Time evolution of the root mean square deviation (RMSD) values of all Cα atoms for the wild-type (black lines) and mutated (red lines) tRNA^Thr^. (**C**) RMSF curves were generated from the backbone atoms for the wild-type (black lines) and mutated (red lines) anticodon stem-loop of tRNA^Thr^. (**D**) Schematic model for the tertiary structure of the anticodon stem-loop for the wild-type (brown) and mutated (blue) tRNA^Thr^.

As shown in Figure 1, the tRNA^Thr^ 15927G>A mutation disrupted the highly conserved base-pairing (28C-42G) of anticodon stem of tRNA^Thr^ (27–29). We therefore hypothesized that the m.15927G>A mutation altered both structure and function of tRNA^Thr^. Functional significances of m.15927G>A mutation were supported by the observations that the lymphoblastoid cell lines bearing the m.15927G>A mutation exhibited the decreased efficiency of aminoacylated tRNA^Thr^, impairment of mitochondrial translation, respiratory deficiency and increasing ROS production (27,30). However, the tissue specific effects of m.15927 G>A mutation-induced mitochondrial dysfunction on the pathological process of coronal atherosclerosis remain elusively. Human umbilical vein endothelial cells (HUVECs) are the most widely used cell models for the study of the regulation of endothelial cell function and the role of the endothelium in the response of the blood vessel wall to stretch, shear forces, and the development of atherosclerotic plaques (31,32). In the present study, we utilized the HUVECs derived cybrids to further investigate the pathophysiology of m.15927G>A mutation. These cybrid cell lines were constructed by transferring mitochondria from lymphoblastoid cell lines derived from a Chinese family carrying the m.15927G>A mutation and from a control individual lacking the mutation but belonging to the same mtDNA haplogroup into mtDNA-less HUVECs, generated by treatment of rhodamine 6G (33–35). The resultant cybrids under these constant nuclear backgrounds allowed us to evaluate the specific effects of m.15927G>A-associated mitochondrial dysfunction on the pathological process of coronal atherosclerosis. First, these cybrid lines were assessed for the effects of the m.15927G>A mutation on tRNA metabolism, mitochondrial translation, respiration, mitochondrial membrane potential, production of reactive oxidative species (ROS) and apoptosis. Then the effects of m.15927G>A mutation-induced alterations on angiogenic properties were investigated by wound healing and tube formation assays.

## MATERIALS AND METHODS

### Cell lines and culture conditions

Immortalized lymphoblastoid cell lines were generated from one affected matrilineal relative (IV-3) of a Chinese family carrying the m.15927G>A mutation and one genetically unrelated Chinese control individual (HZC25) belonging to the same mtDNA haplogroup (B5) but lacking the mutation (Supplementary Table S1) were grown in RPMI 1640 medium with 10% FBS (27,30). Human umbilical vein endothelial cell (HUVEC) line was grown in endothelial basal medium (ScienCell) supplemented with endothelial cell growth supplement (ECGS) and 5% fetal bovine serum (FBS).

To obtain the HUVECs-less-mtDNA lines, HUVECs were treated with 5 μg/ml rhodamine 6G (R6G) under the above media in the presence of 50 μg/ml uridine and 100 mM pyruvate for 7 days (33,34). Transformation by cytoplasts of HUVECs-less-mtDNA lines using enucleated lymphoblastoid cells from one affected subject (IV-3) and one control individual (HZC25) was performed as described elsewhere (33–35). Between 25 and 45 days after fusion, 10–20 presumptive cybrids derived from each donor cell line were isolated, and analyzed for the presence of HUVEC specific m.15833C>T variant to exclude the cybrids carrying the HUVEC mtDNA genetic background. The resultant cybrid clones lacking the m.15833C>T variant were further examined for the presence of m.15927G>A mutation (27) and mtDNA copy numbers (35). Cybrids exhibiting the absence of the m.15927G>A mutation in control clones and its presence in homoplasmic state in all cybrids derived from the mutant cell line were subject to examining the copy number of mtDNA (Supplemental Figure S1). Three cybrid cell clones derived from each donor cell line with similar mtDNA copy numbers were used for the biochemical characterization described below (Supplemental Figure S2). All cybrid cell lines constructed with enucleated lymphoblastoid cell lines were maintained in the same medium as the HUVEC cell line.

### Mitochondrial DNA analysis

Genomic DNA was isolated from the cell lines using a TaKaRa MiniBEST Universal Genomic DNA Extraction Kit. The presence of m.15927G>A mutation and m.15833C>T variant was determined by PCR-amplification of the subject's DNA fragments by the use of oligodeoxynucleotides corresponding to mtDNA at positions 14837–15997 and the Sanger sequence analysis (27). The entire mitochondrial genomes of HUVECs, affected subject (IV-3) and control subject (HZC25) were amplified into 24 overlapping fragments using sets of oligonucleotide primers, as described previously (36). These sequence results were compared with the updated consensus Cambridge sequence (GenBank accession number: NC_012920) (12). The presence and amount of m.15833C>T variant was carried out as follows. In brief, the first PCR segments (1161 bp) were amplified to rule out the co-amplification of possible nuclear pseudogenes. Then, the second PCR products were amplified using the first PCR fragments as template and mismatched oligodeoxynucleotides to introduce *Hind* III restriction site. Equal amounts of various digested samples were then analyzed by electrophoresis through 10% polyacrylamide gel. The proportions of digested and undigested PCR products were determined by the Image-Quant program after ethidium bromide staining (27). The quantification of mtDNA copy numbers from different cybrids was performed by real-time PCR as detailed elsewhere (35).

### MD simulations

#### Simulation systems

The starting coordinates of anticodon stem and loop (ASL) of wild-type tRNA^Thr^ was taken from the crystal structure of Sus scrofa mitochondrial tRNA (Protein Data Bank entry 5AJ3). Then nucleoside bases were substituted according to the mitochondrial tRNA^Thr^ sequence by using Chimera software (37). The coordinaes of G42A mutation mutated base were generated from the wild-type ASL of tRNA^Thr^ through Chimera. Approximately 50 mM NaCl were added to the solvent in addition to the neutralizing Na^+^ or Cl^−^. This leads to a wild-type system of 8658 atoms and a mutant system of 8672 atoms.

#### Simulation protocol

MD simulations were carried out with the Amber14 software (38). The ff03.r1 force field parameters available in Amber14 software. MD trajectories were propagated at a time step of 0.02 fs utilizing the shake algorithm for all hydrogen atoms with a non-bonded cut off of 9.0 Å (39). Energy minimizations were performed to relieve unfavorable contacts, followed with equilibration steps to fully equilibrate the solvent. Each system was equilibrated in the NPT ensemble at 300 K and 1 bar in periodic boundary condition. Positional restraints were first applied on all ALS atoms for 50 fs. After equilibrations, the production simulation was carried out with a time step of 0.02 fs, and each system was run up to 100 ns.

### UV melting assay

UV melting assays were carried out as previously described (24,40). The wild type and mutant tRNA^Thr^ were generated by i*n vitro* transcription by T7 RNA polymerase according to previous protocols (41). The tRNA^Thr^ transcripts were diluted in 50 mM sodium phosphate buffer (pH 7.0) including 50 mM NaCl, 5 mM MgCl_2_ and 0.1 mM EDTA. Absorbance against temperature melting curves were measured at 260 nm with a heating rate of 1°C/min from 25 to 95°C via an Agilent Cary 100 UV Spectrophotometer.

### *In vitro* assay for t^6^A37 modification

The reaction was performed at 30°C in a reaction mixture containing 50 mM Tris–HCl (pH 8.0), 200 mM NaCl, 5 mM MnCl_2_, 1 mM NaHCO_3_, 2 mM DTT, 2.5 mM ATP, 114 μM [^14^C] threonine, 5 μM wild type or 15927 G>A mutant tRNA^Thr^ transcripts and 5 μM yeast Sua5 and Qri7, as detailed previously (42).

### *In vitro* aminoacylation assay

Aminoacylation kinetics were performed at 37°C in a reaction mixture containing 60 mM Tris–HCl (pH 7.5), 10 mM MgCl_2_, 5 mM dithiothreitol (DTT), 0.1 mg/ml bovine serum albumin (BSA), 2.5 mM ATP, 114 μM [^14^C]Thr, 5 μM wild-type tRNA^Thr^ or 15927 G>A mutant and 1 μM human TARS2, as described previously (42).

### Mitochondrial tRNA analysis

Total mitochondrial RNAs were obtained using TOTALLY RNA™ kit (Ambion) from mitochondria isolated from the various cell lines, as described previously (43). Oligodeoxynucleosides used for digoxigenin (DIG) labeled probes of tRNA^Thr^, tRNA^Lys^, tRNA^Glu^, tRNA^His^, tRNA^Met^, tRNA^Ile^, tRNA^Leu(CUN)^, tRNA^Ser(AGY)^ were described as elsewhere (22–24,44,45). DIG-labeled oligodeoxynucleotides were generated by using DIG oligonucleotide Tailing kit (Roche).

For the tRNA Northern blot analysis, 2 μg of total mitochondrial RNA were electrophoresed through a 10% polyacrylamide/8 M urea gel in Tris–borate–EDTA buffer (TBE) after heating the sample at 65°C for 10 min, and then electroblotted onto a positively charged nylon membrane (Roche) for the hybridization analysis with DIG-labeled oligodeoxynucleotide probes. The hybridization and quantification of density in each band were performed as detailed previously (22,44,45).

For the aminoacylation assays, total mitochondrial RNAs were isolated under acid conditions, and two micrograms of total mitochondrial RNAs were electrophoresed at 4°C through an acid (pH 5.0) 10% polycrylamide/8 M urea gel to separate the charged and uncharged tRNA as detailed elsewhere (22,46,47). The gels were then electroblotted onto a positively charged nylon membrane (Roche) for the hybridization analysis with oligodeoxynucleotide probes as described above. Quantification of density in each band was performed as detailed previously (22,46,47).

For the tRNA mobility shift assay, 2 μg of total mitochondrial RNAs were electrophoresed through a 10% polyacrylamide native gel at 4°C in 50 mM Tris-glycine buffer. After electrophoresis, the gels were treated according to the procedure for the tRNA Northern blot analysis described above.

### Western blot analysis

Western blotting analysis was performed as detailed previously (44–46). Twenty micrograms of total proteins obtained from lysed cybrid cells were denatured and loaded on sodium dodecyl sulfate (SDS) polyacrylamide gels. Afterward, the gels were electroblotted onto polyvinylidene difluoride (PVDF) membrane for hybridization. The antibodies used for this investigation were from Abcam [ND1(ab74257), ND3 (ab170681), ND5 (ab92624), CO2 (ab110258), TOM20 (ab56783), cytochrome *c* (ab13575) and Total OXPHOS Human WB Antibody Cocktail (ab110411)], Santa Cruz Biotechnology [ND4 (sc-20499-R) and ND6 (sc-20667)], Proteintech [(CYTB (55090-1-AP), ATP6 (55313-1-AP), ATP8 (26723-1-AP) and β-actin (20536-1-AP)] and Cell Signaling Technology [Caspase-3 (#9664), Caspase-7 (#8438), Caspase-9 (#7237) and PARP (#5625)]. Peroxidase Affini Pure goat anti-mouse IgG and goat anti-rabbit IgG (Jackson) were used as secondary antibodies, and protein signals were detected using the ECL system (CWBIO). Quantification of density in each band was performed as detailed previously (44–46).

### Measurements of oxygen consumption

The rates of oxygen consumption (OCR) in various cell lines were measured with a Seahorse Bioscience XF-96 extracellular flux analyzer (Seahorse Bioscience), as detailed previously (44,48). Cybrid cells were seeded at a density of 2 × 10^4^ cells per well on Seahorse XF96 polystyrene tissue culture plates (Seahorse Bioscience). Inhibitors were used at the following concentrations: Oligomycin (1.5 μM), Carbonyl cyanide 4-trifluoromethoxy-phenylhydrazone (FCCP) (0.8 μM), Antimycin A (1.5 μM) and Rotenone (3 μM).

### Assessment of mitochondrial membrane potential

Mitochondrial membrane potential was assessed with JC-10 Assay Kit-Flow Cytometry (Abcam) following general manufacturer's recommendations with some modifications, as detailed elsewhere (44,49). In brief, ∼2 × 10^6^ cells of each cybrid cell line were harvested, resuspended in 200 μl 1 × JC-10 Assay Buffer and then incubated at 37°C for 30 min. Alternatively, harvested cells were preincuated with 10 μM of FCCP for 30 min at 37°C prior to staining with JC-10 dye. After washing with PBS twice, cells were resuspended in 200 μl PBS. The fluorescent intendities for both J-aggregates and monomeric forms of JC-10 were measured at Ex/Em = 490/530 and 490/590 nm with DB-LSR II flow cytometer system (Beckton Dickson, Inc.).

### Measurement of mitochondrial ROS production

The levels of mitochondrial reactive oxygen species (ROS) generation were determined using MitoSOX assay as detailed previously (50–52). Briefly, approximate 2 × 10^6^ cells of each cell line were harvested, resuspended in 5 μM MitoSOX reagent working solution and then incubated at 37°C for 20 min. After washing with PBS twice, cells were resuspended in PBS in the presence of 2 mM freshly prepared H_2_O_2_ and 2% FBS and then incubated at room temperature for another 45 min. Cells were further washed with PBS and resuspended with 1 ml of PBS with 0.5% paraformaldehyde. Samples with or without H_2_O_2_ stimulation were analyzed by BD-LSR II flow cytometer system (Beckton Dickson, Inc.), with an excitation at 488 nm and emission at 529 nm. Ten thousand events were analyzed in each sample.

### Immunofluorescence analysis

Immunofluorescence experiments were performed as described elsewhere (35,52). Cells were cultured on cover glass slips (Thermo Fisher), fixed in 4% formaldehyde for 15 min, permeabilized with 0.2% Triton X-100, blocked with 5% Fetal Bovine Serum (FBS) for 1 h, and immunostained with cytochrome *c* antibody overnight at 4°C. The cells were then incubated with Alex Fluor 488 goat anti-mouse IgG (H+L) (Thermo Fisher), stained with MitoTracker Red (Invitrogen) for 20 min and with 4′,6-diamidino-2-phenylindole (DAPI; Invitrogen) for 15 min, and mounted with Fluoromount (Sigma-Aldrich). Cells were examined using a confocal fluorescence microscope (Olympus Fluoview FV1000, Japan) with three lasers (Ex/Em = 550/570, 492/520 and 358/461 nm).

### Wound healing assay

Wound healing assays were performed as described elsewhere (53). In brief, mutant and control cybrid cells were seeded on 24-well plates and cultured until confluent. Then, a scratch wound with a 200 μl pipet tip was made across the middle of the cell monolayer at the length of the plate. The cells were then incubated with serum free medium. Images were taken by Leica Microsystems immediately after scratch and the same windows were imaged again 24 h later. The rate of cell migration was calculated with NIH ImageJ analyzer as the average percent wound closure from at least three independent experiments.

### Tube formation assay

A tube formation assay using growth factor-reduced Matrigel was carried out as detailed elsewhere (54,55). Briefly, mutant and control cybrids were cultured as above and washed with PBS three times and detached with 0.05% trypsin/EDTA. After centrifugation with 1000 rpm for 5 min, cell pellets were washed again with PBS and counted with a hemocytometer. An aliquot of Matrigel (Matrigel Matrix, Corning) was thawed at 4°C overnight, dispersed onto prechilled 12-well plates (200 μl per well) and allowed to polymerize for 30 min at 37°C. 1 × 10^5^ cells were resuspended with medium and loaded on top of Matrigel. Following incubation at 37°C for 12 h, each well was analyzed directly for tube formation under an inverted microscope (Leica Microsystems, Germany). Under a microscope with 40× phase contrast, tubules in each field were imaged. The tube formation parameters including total tube length, number of nodes, number of junctions, number of master segments, total master segments length, number of meshs, total mesh area, number of branches and total branch length were assessed by Angiogenesis Analyzer for ImageJ (http://imagej.nih.gov/ij/macros/toolsets/Angiogenesis%20Analyzer.txt) from at least nine random fields for each cell line.

### Statistical analysis

Statistical analysis was performed by the unpaired, two-tailed Student's t-test contained in Microsoft Office Excel (version 2013). *P* indicates the significance, according to the t-test, of the difference between mutant and control mean. Differences were considered significant at a *P* < 0.05.

## RESULTS

### MD simulation analyses

We performed the molecular dynamics simulation to examine whether the m.15927G>A mutation perturbs the structure of tRNA^Thr^. This method has been widely used for evaluating structural impact of diseasing-causing mutations (38,56). Based on the rational initial structure, the ASL (17 nt) of both wild-type and mutated tRNA^Thr^ were evaluated by 100-ns all-atom molecular dynamics simulations. As shown in Figure 1B, root mean square deviation (RMSD) curve of the mutated ASL fluctuated more heavily than that of the wild-type counterpart, suggesting that the mutated ASL exhibited more unstable than its wild-type counterpart. Furthermore, we carried out root mean square fluctuation (RMSF) analysis on the two trajectories to analyze the mobility of ASL. As shown in Figure 1C, the RMSF values of A42 in mutated ASL were much higher than that of the wild-type form, further supporting that the mutant ASL was more unstable than that of wild-type counterpart. These data strongly indicated that the disruption of the 28C-42G base-pairing of anticodon stem of tRNA^Thr^ accounted for the less stability of the tRNA^Thr^ structure (Figure 1D).

### Altered conformation and stability of tRNA^Thr^

**Figure 2.**
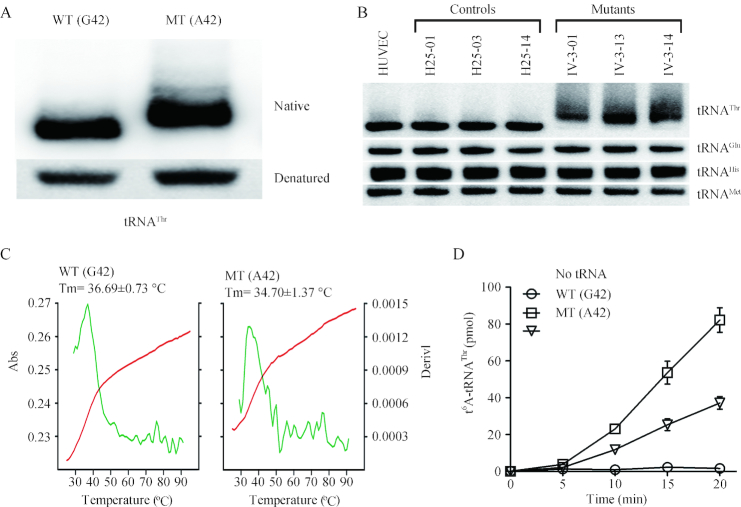
*In vitro* analysis for the conformation, stability and t^6^A37 modification of tRNA^Thr^. (**A**) Assessment of conformation changes by PAGE analysis under denaturing and native conditions. The transcripts of wild-type (WT) and mutated (MT) tRNA^Thr^ were electrophoresed through native or denaturing polyacrylamide gel stained with ethidium bromide. (**B**) Northern blot analysis of tRNAs under native conditions. Two micrograms of total mitochondrial RNA from various cell lines were electrophoresed through native polyacrylamide gel, electroblotted, and hybridized with DIG-labeled oligonucleotide probes specific for the tRNA^Thr^, tRNA^Glu^, tRNA^His^, and tRNA^Met^, respectively. (**C**) Melting profiles of WT and MT tRNA^Thr^ transcripts measured at 260 nm with a heating rate of 1°/min from 25 to 95° (red curves). First derivative (d*A*/d*T*) against temperature curves were shown to highlight the *T*_m_ value transitions (green curves). (**D**) *In vitro* assay for t^6^A37 modification. The unmodified human mitochondrial wild type (G42) and mutant (A42) tRNA^Thr^ were generated from *in vitro* transcription. The unmodified tRNA transcripts were incubated with yeast Sua5 and Qri7 in the presence of [^14^C] threonine. Samples were withdrawn and stopped after 5, 10, 15 or 20 min, respectively. The relative modification efficiency was calculated from the initial phase of the reaction. The calculations were based on three independent determinations. Graph shows the results of a representative experiment.

As shown in Figure 2A, electrophoretic patterns showed there the mutant (A42) tRNA^Thr^ transcript migrated slower than the wild type (G42) tRNA^Thr^ transcript under the native condition. However, there were no difference of migration pattern between wild-type (G42) and mutant (A42) tRNA^Thr^ transcripts under denaturing condition.

To test if the m.15927G>A mutation affected the conformation of tRNA^Thr^*in vivo*, 2 μg of total mitochondrial RNAs isolated from mutant and control cybrids and HUVECs were electrophoresed through 10% native polyacrylamide gel in Tris-glycine buffer and then electroblotted onto a positively charged nylon membrane for hybridization analysis with DIG-labeled oligodeoxynucleotide probes for tRNA^Thr^, tRNA^Glu^, tRNA^His^ and tRNA^Met^ respectively. As shown in Figure 2B, electrophoretic patterns showed that the tRNA^Thr^ in three mutant cybrid cell lines carrying the m.15927G>A mutation migrated much slower than those of three control cybrid cell lines lacking this mutation. Strikingly, long smearing bands were observed in the mutant tRNA^Thr^.

We then measured the melting temperature (Tm) by calculating the derivatives of the absorbance against a temperature curve. As shown in Figure 2C, the *T*_m_ values of wild-type (G42) and mutant (A42) transcripts were 36.69 and 34.70°C, respectively. These data suggested that the m.15927G>A mutation perturbed the conformation and stability of tRNA^Thr^.

### Marked decrease in t^6^A37 modification of tRNA^Thr^*in vitro*

*N* ^6^-threonylcarbamoyladenosine (t^6^A) is universally conserved modification present at position 37 of tRNAs, including human mitochondrial tRNA^Thr^ (14). The posttranscriptional modification could stabilize the anticodon loop structure and enhance tRNA binding to the A-site codon, as well as maintain the efficiency and accuracy of translation (57). To assess if the m.15927G>A mutation caused the deficient t^6^A modification, we measured the level of the t^6^A37 modification of both mutant and wild-type tRNA^Thr^ transcripts, as well as a control without tRNA addition, catalyzed by yeast Sua5 and Qri7. As shown in Figure 2D, 55% decrease in the level of t^6^A modification was observed in the mutant tRNA^Thr^ transcript (A42), as compared with that of wild-type tRNA^Thr^ (G42).

### Reductions in the steady-state level of tRNA^Thr^

**Figure 3.**
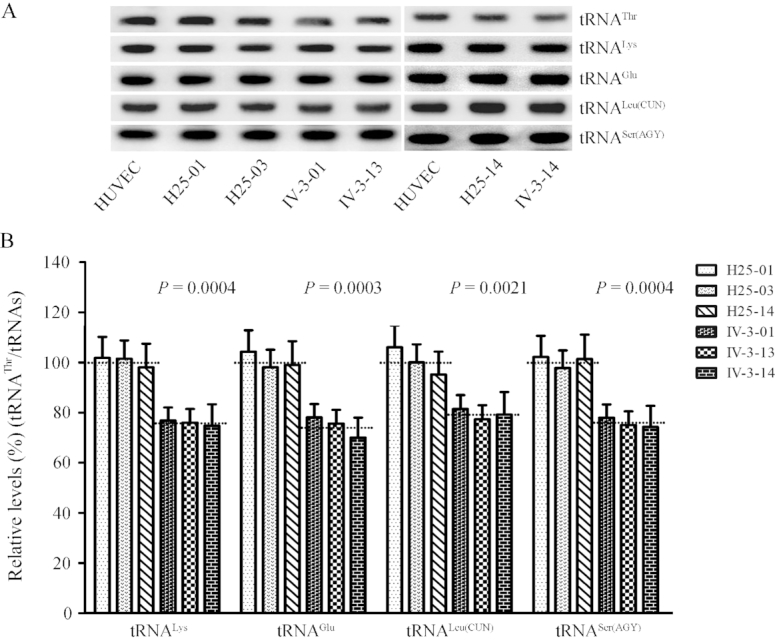
Northern blotting analysis of mitochondrial tRNA under denaturing conditions. (**A**) Two micrograms of total mitochondrial RNA from various cell lines were electrophoresed through a denaturing polyacrylamide gel, electroblotted, and hybridized with DIG-labeled oligonucleotide probes specific for the tRNA^Thr^, tRNA^Lys^, tRNA^Glu^, tRNA^Leu(CUN)^ and tRNA^Ser(AGY)^, respectively. (**B**) Quantification of tRNA levels. The average tRNA^Thr^ content per cell, normalized to the average content per cell of tRNA^Lys^, tRNA^Glu^, tRNA ^Leu(CUN)^, and tRNA^Ser(AGY)^ in three cybrid cell lines derived from one affected subject (IV-3) carrying the m.15927 G > A mutation and three cybrid cell lines derived from one Chinese control individual (HZC25) belonging to the same mtDNA haplogroup (B5) but lacking this mutation. The values for the cybrid cell lines are expressed as percentages of the average value for the control cell lines. The calculations were based on three independent determinations. The error bars indicate two standard errors of the means, the horizontal dashed lines represent the average value for each group. *P* indicates the significance, according to the *t*-test, of the differences between mutant and control cybrid cell lines.

To examine whether the m.15927G>A mutation affects the *in vivo* metabolism of tRNA^Thr^, we subjected total mitochondrial RNA from HUVEC and cybrid cell lines to Northern blots and hybridized them with DIG-labeled oligodeoxynucleotide probes for tRNA^Thr^, tRNA^Lys^, tRNA^Leu(CUN)^, tRNA^Ser(AGY)^ as representatives of the heavy (H)-strand transcription unit and tRNA^Glu^ derived from the light (L)-strand transcription respectively (58,59). As shown in Figure 3A, the amount of tRNA^Thr^ in three mutant cybrid cell lines was decreased, compared with those in three control cybrid cell lines. The average levels of tRNA^Thr^ in the mutant cybrid cell lines were 75.5% (*P* = 0.0004), 74.9% (*P* = 0.0004), 78.3% (*P* = 0.0016) and 76.4% (*P* = 0.0006) of average values of three control cell lines after normalization to tRNA^Lys^, tRNA^Glu^, tRNA^Leu(CUN)^ and tRNA^Ser(AGY)^, respectively (Figure 3B).

### Impaired aminoacylation of tRNA^Thr^

**Figure 4.**
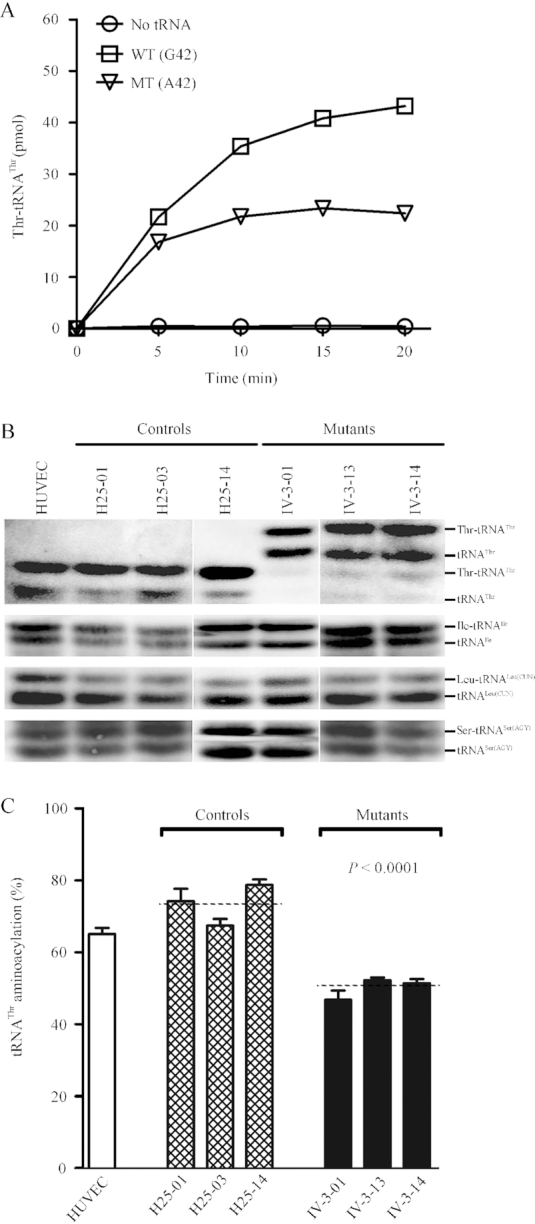
Aminoacylation assays. (**A**) *In vitro* aminoacylation of transcribed human mitochondrial mutant and wild type tRNA^Thr^. The unmodified human mitochondrial wild type (G42) and mutant (A42) tRNA^Thr^ were incubated with human TARS2 in the presence of [^14^C] threonine. Samples were withdrawn and stopped after 5, 10, 15 or 20 min, respectively. The relative aminoaylation efficiency was calculated from the initial phase of the reaction. The calculations were based on three independent determinations. Graph shows the results of a representative experiment. (**B**) *In vivo* aminoacylation assays. Two micrograms of total mitochondrial RNA purified from various cell lines under acid conditions were electrotrophoresed at 4°C through an acid (pH 5.0) 10% polycrylamide/8M urea gel, electroblotted and hybridized with a DIG-labeled oligonucleotide probe specific for the tRNA^Thr^. The blots were then stripped and rehybridized with probes specific for the tRNA^Ile^, tRNA^Leu(CUN)^ and tRNA^Ser(AGY)^, respectively. (**C**) The proportion of aminoacylated tRNA^Thr^ in the mutant, control cybrid cell lines and HUVECs. The calculations were based on three independent determinations. Graph details and symbols are explained in the legend to Figure 3.

The central role of tRNA is the generation of aminoacyl-tRNA, catalyzed by its cognate aminoacyl-tRNA synthetase, to supply the materials of protein biosynthesis (47). To understand the effect of m.15927G>A mutation on the charging capacity of tRNA^Thr^, we assessed the aminoacylation properties of wild-type and mutant tRNA^Thr^ transcripts, as well as a control without tRNA addition, catalyzed by human TARS2. As shown in Figure 4A, the efficiencies of aminoacylated tRNA^Thr^ in the mutant transcript (A42) reflected 48.2% decrease, relative to the average values of wild-type tRNA^Thr^ transcript (G42).

The aminoacylation capacities of tRNA^Thr^, tRNA^Ile^, tRNA^Leu(CUN)^ and tRNA^Ser(AGY)^ in various cybrid cell lines were further examined by the use of electrophoresis in an acidic polyacrylamide/urea gel system to separate uncharged tRNA species from the corresponding charged tRNA, electroblotting and hybridizing with tRNA probes described elsewhere (46,47). Electrophoretic patterns showed that either charged (upper band) or uncharged (lower band) tRNA^Thr^ in cell lines carrying the m.15927G>A mutation migrated much slower than those of cell lines lacking this mutation. However, there were no obvious differences in electrophoretic mobility of tRNA^Ile^, tRNA^Leu(CUN)^ and tRNA^Ser(AGY)^ between the cell lines carrying the m.15927G>A mutation and cell lines lacking this mutation (Figure 4B). Notably, the proportions of aminoacylated tRNAs in the mutant cybird cells were 50.3%, 51.9%, 36.2% and 54% in the tRNA^Thr^, tRNA^Ile^, tRNA^Leu(CUN)^ and tRNA^Ser(AGY)^ respectively, while 73.7% of tRNA^Thr^, 53% of tRNA^Ile^, 36.6% of tRNA^Leu(CUN)^ and 52.9% of tRNA^Ser(AGY)^ were aminoacylated in the control cybrid cells, respectively. The efficiencies of aminoacylated tRNA^Thr^ in these mutant cybrid cell lines carrying the m.15927G>A mutation reflected 31.7% reduction, ranged from 28.9% to 36.3%, relative to the average values of control cybrid cell lines (*P* < 0.0001) (Figure 4C). However, the levels of aminoacylation in tRNA^Ile^, tRNA^Leu(CUN)^ and tRNA^Ser(AGY)^ in mutant cell lines were comparable with those in control cell lines (Supplemental Figure S3).

### Reduction in the level of mitochondrial proteins

**Figure 5.**
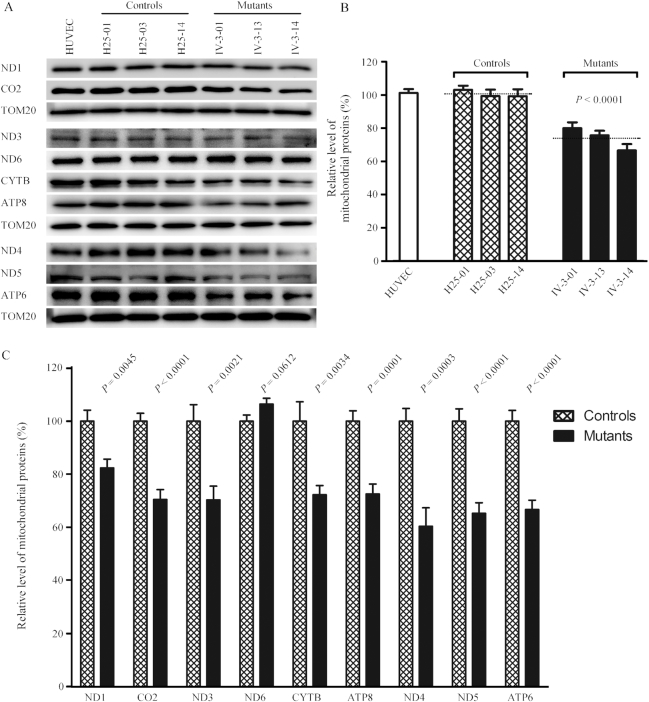
Western blot analysis of mitochondrial proteins. (**A**) Five micrograms of total mitochondrial proteins from various cell lines were electrophoresed through a denaturing polyacrylamide gel, electroblotted, and hybridized with nine respiratory complex subunits in mutant, control cybrid cell lines and HUVECs with TOM20 as a loading control. ND1, ND3, ND4, ND5 and ND6, indicate subunits 1, 3, 4, 5 and 6 of the reduced nicotinamide-adenine dinucleotide dehydrogenase; CYTB, apocytochrome *b*; CO_2_, subunit II of cytochrome *c* oxidase; ATP6 and ATP8, subunit 6 and 8 of the H^+^-ATPase. (**B**) Quantification of total mitochondrial protein levels. The levels of mitochondrial proteins in HUVEC and six cybrid cell lines were determined as described elsewhere (22–24). The values for the mutant cybrid cell lines are expressed as percentages of the values for the control cell lines. The calculations were based on three independent determinations. (**C**) Quantification of levels of 9 polypeptides. The levels of ND1, ND3, ND4, ND5, ND6, CYTB, CO2, ATP6 and ATP8 in HUVEC and six cybrid cell lines were determined as described elsewhere (22–24). Graph details and symbols are explained in the legend to Figure 3.

In order to investigate whether the m.15927G>A mutation impaired mitochondrial translation, a Western blot analysis was carried out to examine the levels of 9 mtDNA encoded respiratory complex subunits in mutant and control cybrid cell lines with a nuclear encoding mitochondrial protein TOM20 as loading control. As shown in Figure 5A, the levels of ND1, ND3, ND4, ND5 (subunits 1, 3, 4, 5 of NADH dehydrogenase), CO2 (subunit II of cytochrome *c* oxidase); CYTB (apocytochrome *b*) and ATP6 and ATP8 (subunits 6 and 8 of H^+^-ATPase) exhibited variable reductions in mutant cybrid cell lines, whereas the levels of ND6 (subunit 6 of NADH dehydrogenase) in the mutant cybrid cell lines comparable with those in control cybrid cells. The average overall levels of nine mitochondrial translation products in the three mutant cybrid cell lines were 73% (*P* < 0.0001), relative to the mean value measured in the control cybrid cell lines (Figure 5B). Notably, the average levels of ND1, ND3, ND4, ND5, ND6, CO2, CYTB, ATP6 and ATP8 in the mutant cybrid cells were 82.4%, 70.3%, 60.4%, 65.2%, 106.4%, 70.5%, 72.3%, 66.7% and 72.6% of the average values of control cells, respectively (Figure 5C). However, the levels of polypeptide synthesis in mutant cells, relative to those in control cells, showed no significant correlation with either the number of codons or the proportion of threonine residues (Supplementary Table S2).

**Figure 6.**
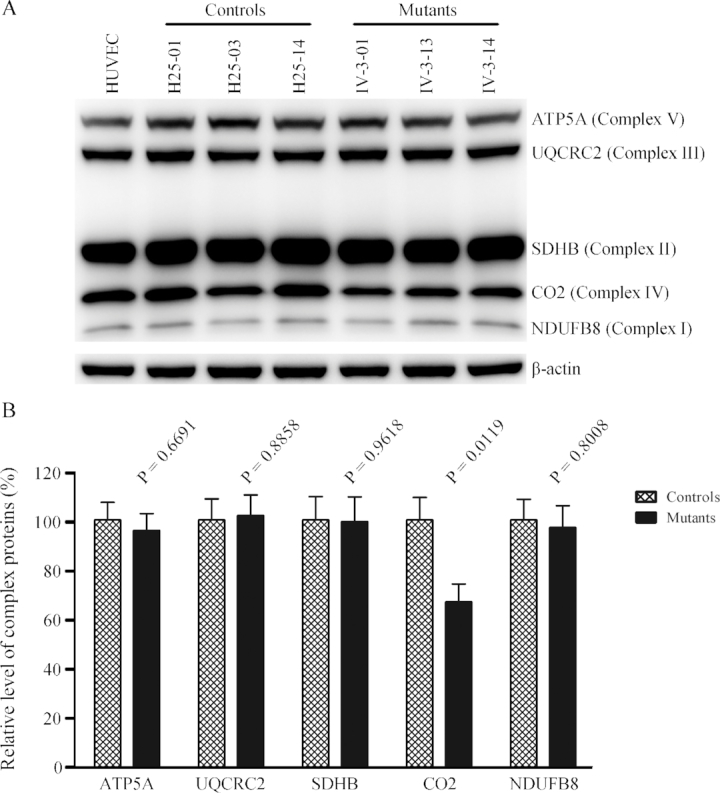
Western blot analysis of OXPHOS subunits. (**A**) Twenty micrograms of total cellular proteins from various cell lines were electrophoresed through a denaturing polyacrylamide gel, electroblotted and hybridized with antibody cocktail specific for subunits of each OXPHOS complex and with β-actin as a loading control. (**B**) Quantification of the levels of ATP5A, UQCRC2, SDHB, CO_2_ and NDUFB8 in mutant and control cell lines were determined as described elsewhere (52). Graph details and symbols are explained in the legend to Figure 3.

We then examined the levels of five subunits of oxidative phosphorylation (OXPHOS) complexes in control and mutant cybrid cell lines by western blot analysis using the total OXPHOS human antibodies cocktail containing antibodies for mtDNA encoded subunit CO_2_ of cytochrome *c* oxidase and four other polypeptides (NDUFB8 of NADH:ubiquinone oxidoreductase; SDHB of succinate ubiquinone oxidoreductase; UQCRC2 of ubiquinol cytochrome *c* reductase and ATP5A of H^+^-ATPase) encoded by nuclear genes. As shown in Figure 6, the average level of CO2 in the mutant cybrid cells were 66.7% (*P* = 0.0119) of control cell lines. In contrast, the levels of the nuclear genes encoded subunits NDUFB8, SDHB, UQCRC2 and ATP5A in mutant cybrid cell lines were comparable with those in control cybrid cell lines.

### Respiration deficiency

**Figure 7.**
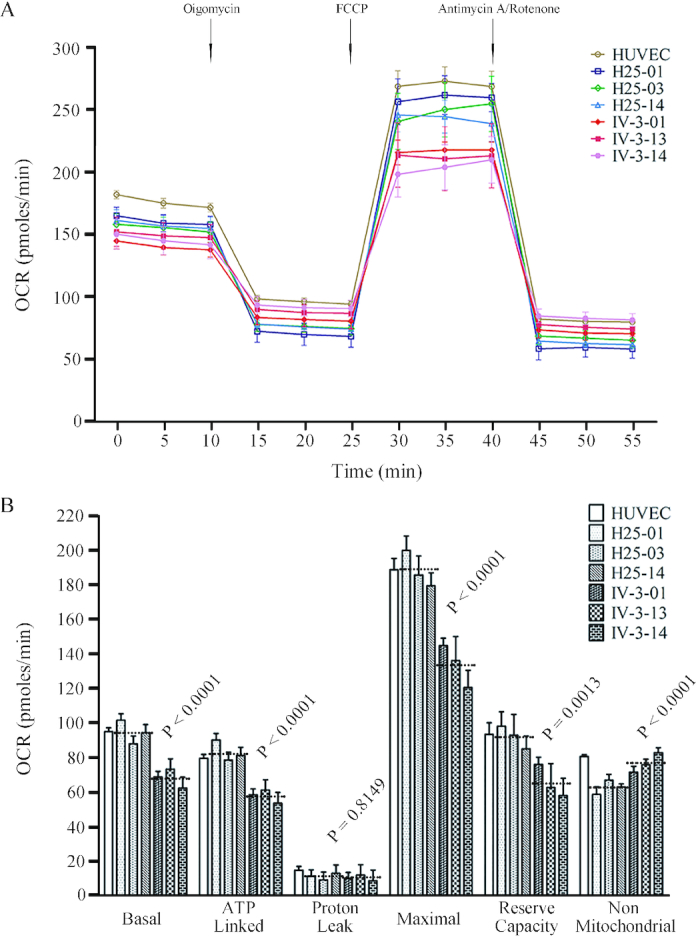
Respiration assays. (**A**) Analysis of O_2_ consumption in the various cell lines using different inhibitors. The rates of oxygen consumption (OCR) were first measured on 2 × 10^4^ cells of each cell line under basal conditions and then with sequential additions of oligomycin (1.5 μM), FCCP (0.8 μM), rotenone (3.0 μM), and antimycin A (1.5 μM) at the indicated times to determine different parameters of mitochondrial functions. (**B**) Graphs presented the basal OCR, ATP-linked OCR, proton leak OCR, maximal OCR, reserve capacity OCR and non-mitochondrial OCR in HUVEC, mutant and control cybrid cell lines. Basal OCR was determined as OCR before oligomycin minus OCR after rotenone/antimycin A. ATP-linked OCR was determined as OCR before oligomycin minus OCR after oligomycin. Proton leak OCR was determined as basal OCR minus ATP-linked OCR. Maximal OCR was determined as the OCR after FCCP minus non-mitochondrial OCR. Reserve capacity OCR was defined as the difference between maximal OCR after FCCP minus basal OCR. Non-mitochondrial OCR was determined as the OCR after rotenone/antimycin A treatment. The average of 6 determinations for each cell line is shown. Graph details and symbols are explained in the legend to Figure 3.

To evaluate whether the m.15927G>A mutation affects cellular bioenergetics, we measured the OCRs of various mutant and control cybrid cell lines using a Seahorse Bioscience XF-96 Extracellular Flux Analyzer. As shown in Figure 7, the basal OCR in the mutant cybrid cell lines was 72.1% (*P* < 0.0001) relative to the mean value measured in the control cybrid cell lines. To further investigate which of the enzyme complexes of the respiratory chain was affected in the mutant cybrid cell lines, OCR was measured after the sequential addition of oligomycin (to inhibit the ATP synthase), FCCP (to uncouple the mitochondrial inner membrane and allow for maximum electron flux through the electron transfer chain), antimycin A (to inhibit complex III) and rotenone (to inhibit complex I) (60). The difference between the basal OCR and the drug-insensitive OCR yields the amount of ATP linked OCR, proton leak OCR, maximal OCR, reserve capacity OCR and non-mitochondrial OCR. The ATP linked OCR, proton leak OCR, maximal OCR, reserve capacity OCR and non-mitochondrial OCR in mutant cybrid cell lines were 69.4% (*P* < 0.0001), 99.9% (*P* = 0.8149), 71.1% (*P* < 0.0001), 71.3% (*P* = 0.0013) and 122.2% (*P* < 0.0001) relative to the mean value measured in the control cybrid cell lines respectively (Figure 7B).

### Decrease in mitochondrial membrane potential

**Figure 8.**
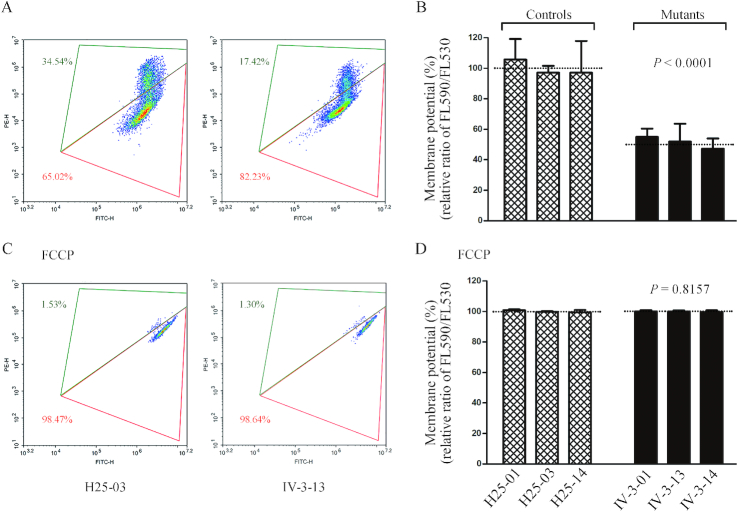
Mitochondrial membrane potential analysis. The mitochondrial membrane potential (ΔΨm) was measured in mutant and control cybrid cell lines by BD-LSR II flow cytometer system using a fluorescence probe JC-10 assay system. The ratio of the Ex/Em = 488/590 nm and 488/525 nm fluorescence intensities (FL590/FL525) was recorded to determine the ΔΨm level of each sample. Flow cytometry images of H25-03 and IV-3-13 without (**A**) and with (**C**) FCCP. Relative ratio of JC-10 fluorescence intensities at Ex/Em = 490/530 nm and 490/590 nm in (**B**) absence and (**D**) presence of 10 μM of FCCP. Graph details and symbols are explained in the legend to Figure 3.

The mitochondrial membrane potential (ΔΨm) generated by proton pumps (Complexes I, III and IV) is an essential component in the process of energy storage during oxidative phosphorylation. Together with the proton gradient (ΔpH), ΔΨm forms the transmembrane potential of hydrogen ions which is harnessed to make ATP (49). As shown in Figure 8, the average levels of the ΔΨm in three mutant cybrids carrying the m.15927 G>A mutation ranged from 47.3% to 55%, with an average of 51.4% (*P* < 0.0001) of the mean value measured in three control cybrids. In contrast, the levels of ΔΨm in mutant cybrids in the presence of FCCP were comparable with those measured in the control cybrid cell lines.

### The increase of mitochondrial ROS production

**Figure 9.**
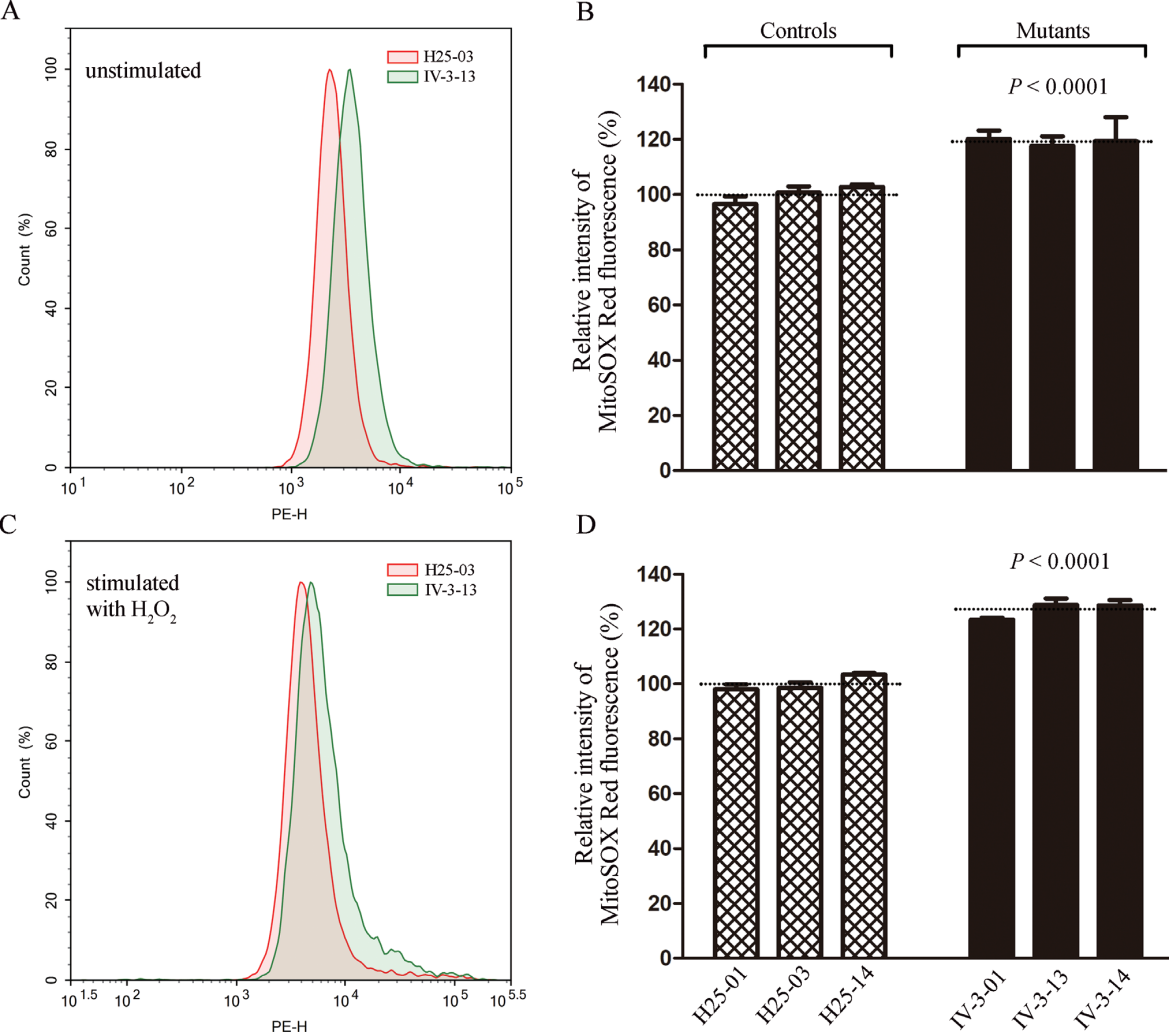
Measurement of mitochondrial ROS. Ratio of geometric mean intensity between levels of the ROS generation in the vital cells with or without H_2_O_2_ stimulation. The rates of mitochondrial ROS production in three mutant cybrid cell lines, three control cell lines and HUVECs, were analyzed by BD-LSR II flow cytometer system using MitoSOX Red Mitochondrial Superoxide Indicator. Flow cytometry histogram showing MitoSOX-Red fluorescence of H25-03 (red) and IV-3-13 (green) without (**A**) or with (**C**) H_2_O_2_ stimulation. The relative ratios of fluorescence intensity (stimulated versus unstimulated with H_2_O_2_) was calculated: (**B**) and (**D**). Graph details and symbols are explained in the legend to Figure 3.

Mitochondria ROS play the critical role in the physiological consequences (61). The levels of mitochondrial ROS generation in three mutant cybrids cell lines carrying the m.15927G>A mutation and three control cybrid cell lines lacking the mutation were determined using MitoSOX assay via flow cytometry under normal conditions and then following H_2_O_2_ stimulation (23,44,50). Geometric mean intensity was recorded to measure the production rate of ROS of each sample. As shown in Figure 9A and B, the levels of ROS generation in the mutant cybrid cell lines carrying the m.15927G>A mutation ranged from 117.6% to 120.2%, with an average of 119.1% (*P* < 0.0001) of the mean value measured in control cybrid cell lines under unstimulated conditions. As illustrated in Figure 9C and D, the levels of ROS generation in mutant cybrid cell lines varied from 123.4% to 128.7%, with an average of 126.9% (*P* < 0.0001) of the mean value measured in the control cybrid cell lines under stimulation conditions.

### Promoting apoptosis

**Figure 10.**
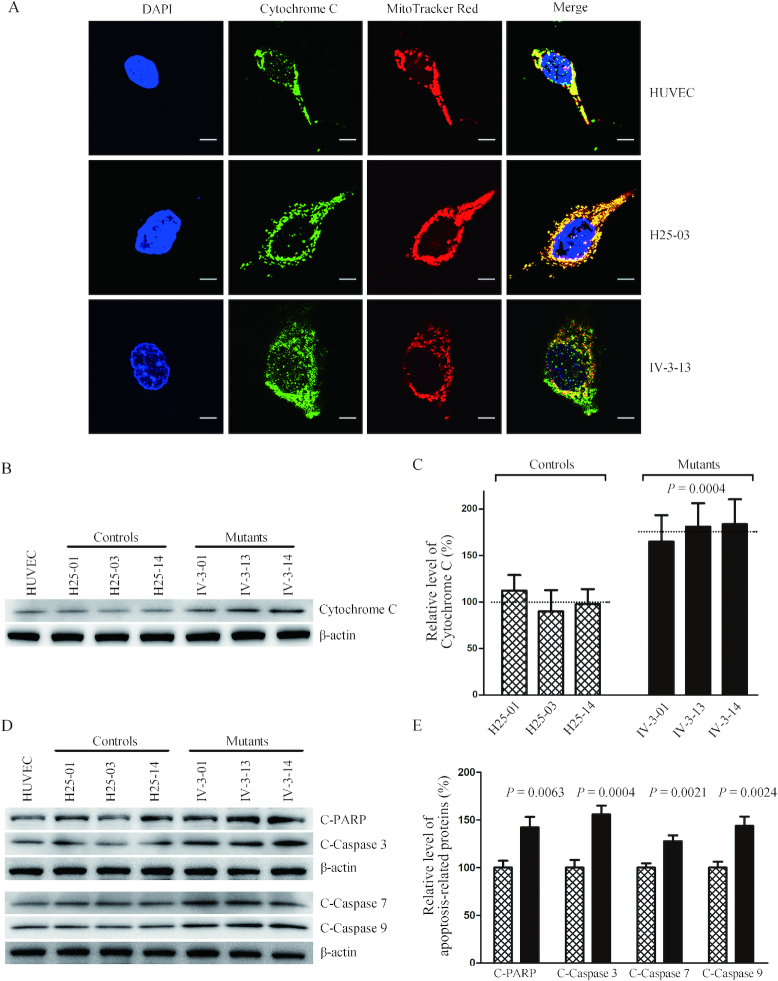
Analysis of apoptosis. (**A**) The distributions of cytochrome *c* from control cybrid cell line (H25-03), mutant cybrid cell line (IV-3-13) and HUVECs were visualized by immunofluorescent labeling with cytochrome *c* antibody conjugated to Alex Fluor 488 (green) analyzed by a confocal fluorescence microscope. Mitotracker Red-stained mitochondria and DAPI-stained nuclei were identified by red and blue fluorescence respectively. (**B**) Twenty micorgrams of total proteins from various cell lines were electrophoresed through a denaturing polyacrylamide gel, electroblotted, and hybridized with cytochrome *c* antibody with β-actin as a loading control. (**C**) Quantification of cytochrome *c* levels. The levels of cytochrome *c* in control and mutant cell lines were determined as described elsewhere (53). The calculations were based on three independent determinations in each cell line. (**D**) Twenty micrograms of total proteins from various cell lines were electrophoresed through a denaturing polyacrylamide gel, electroblotted, and hybridized with cleaved PARP, Caspases 3, 7 and 9 antibodies with β-actin as a loading control. (**E**) Quantification of four apoptosis-activated proteins. The levels of Caspases 9, 3, 7 and PARP in various cell lines were determined as described elsewhere (52). The calculations were based on three independent determinations in each cell line. Graph details and symbols are explained in the legend to Figure 3.

To examine if the m.15927 G>A mutation affects the apoptotic process, we examined the apoptotic state of mutant and control cybrid cell lines by using both immunofluorescent staining and Western blot analysis. In particular, release of cytochrome *c* is a critical apoptotic event at the level of the mitochondria and can in turn promote programmed death by activating apoptotic cascades (62). As shown in Figure 10A, the immunofluorescence patterns of double-labeled cells with rabbit monoclonal antibody specific for the cytochrome *c* and specific dye for mitochondria MitoTracker Red revealed markedly increased levels of cytosolic cytochrome *c* in the mutant cells, compared to control cells. The levels of cytochrome *c* in mutant and control cell lines were further evaluated by western blotting analysis (Figure 10B). As shown in Figure 10C, the levels of cytochrome *c* in three mutant cell lines ranged from 164.9% to 183.7%, with an average of 176.5% (*P* = 0.0004), relative to the average values in three control cell lines. Furthermore, we examined the levels of four apoptosis-activated proteins (Caspases 9, 3, 7 and PARP) in mutant and control cell lines by western blot analysis (63), with marked increase in the mutant cell lines (Figure 10D). In particular, the levels of Caspases 3, 7, 9 and PARP in the mutant cell lines were 142.2%, 155.8%, 127.7% and 143.7% of the average values measured in the control cell lines, respectively (*P* = 0.0004–0.0063).

### Reduced wound healing properties

**Figure 11.**
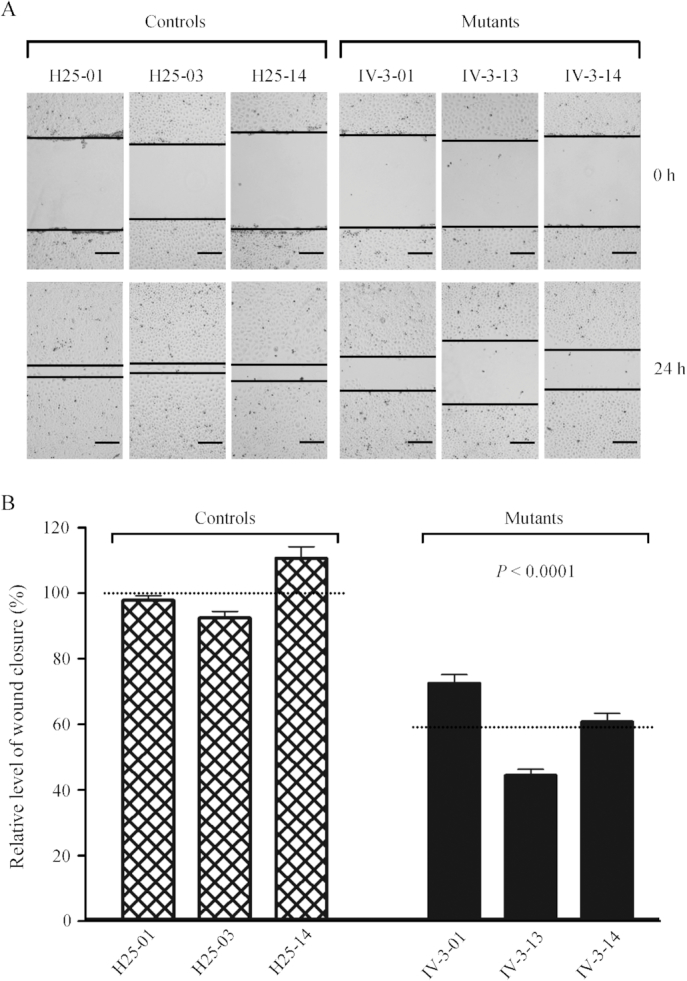
Wound healing assay. (**A**) Representative images of wound healing assay for three control and three mutant cybrid cell lines. Photographs were taken directly immediately after scratch and 24 h after wounding. The outlines show the gap area of the wounds. Scale bars, 100 μm. (**B**) Quantification of wound healing rates. Quantitative measurement of cell migration was performed at 24 h after wounding as described elsewhere (53). The calculations were based on 3–4 independent determinations in each cell line. Graph details and symbols are explained in the legend to Figure 3.

Wound healing assays with live-cell microscopy using mutant and control cybrids derived from HUVECs were performed to analyze the effect of the m.15927G>A mutation on angiogenesis and wound regeneration. The healing of wound is a complex process that involves four distinct, but overlapping phases: hemostasis, inflammation, remodeling/granulation tissue formation and re-epithelialization (64). Inflammation and angiogenesis are two fundamental physiological conditions implicated in this process. In the present study, mutant and control cybrids were wounded with a scratch and incubated with serum free medium for 24 h to impair healing and then visualized by an optical microscopy. As illustrated in Figure 11A, the mutant cybrid cell lines bearing the m.15927G>A mutation exhibited lower wound healing cell migration than those in control cybrid cell lines after culturing cell for 24 h after wounding. The levels of wound closure in the mutant cybrid lines derived from 44% to 72.1%, with an average of 59.1% (*P* < 0.0001), compared with the average values of control cybrid lines (Figure 11B).

### Altered angiogenesis

**Figure 12.**
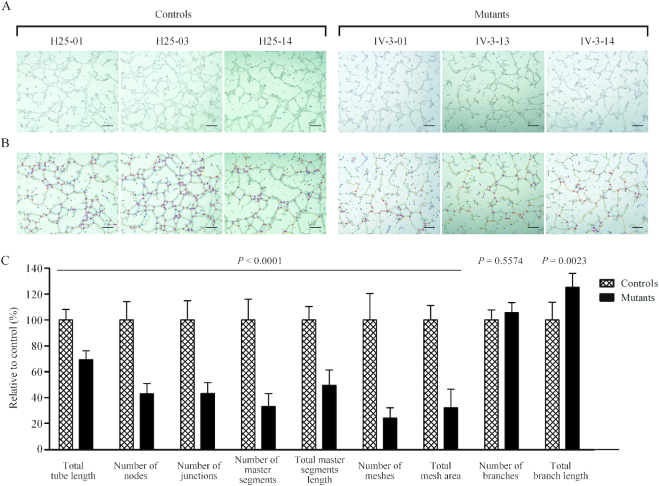
Tube formation Assay. Cells (1 × 10^5^) from three control and three mutant cybrids cells were grown and resuspended with medium and loaded on top of Matrigel. Following incubation at 37°C for 12 h, each well was analyzed directly for tube formation under an inverted microscope. (**A**) Representative light photomicrographs of tube formation for three control cybrids and three mutant cybrids after plating onto Matrigel for 16 h. (**B**) The photomicrographs were analyzed using Angiogenesis Analyzer (ImageJ), different structures of the tubule network are labeled with different colors representing its own feature: master segments (orange), meshes (sky blue), nodes surrounded by junction symbol (red surrounded by blue) and branches (green). (**C**) Quantitative analysis of specific parameters of capillary tube formation with the Angiogenesis Analyzer for ImageJ. The parameters include total tube length, number of nodes, number of junctions, number of master segments, total master segments length, number of meshs, total mesh area, number of branches and total branch length. Data represent an average of nine fields of each cell line. Scale bars, 500μm. Graph details and symbols are explained in the legend to Figure 3.

To further investigate the effect of m.15927G>A mutation on angiogenesis, we carried out the tube formation assay, a rapid and quantitative method for evaluating angiogenesis (55). The mutant and control HUVECs-derived cybrids were cultured in the presence of growth factor-reduced Matrigel, an extract of endothelial basement membrane for 16 h, to induce the differentiation and tube-like structure formation. As shown in Figure 12A, mutant and control cybrid cells as well as HUVECs gradually stretched, and connected each other into cords and network structure, forming luminal structures of various sizes and shapes after loading on the top of Matrigel. The captured images were analyzed by ImageJ with the Angiogenesis Analyzer plugin (http://imagej.nih.gov/ij/macros/toolsets/Angiogenesis%20Analyzer.txt) to quantify different parameters, such as master segments (orange), meshes (sky blue), nodes surrounded by junctions symbol(red surrounded by blue) and branches (green) (Figure 12B). As illustrated in Figure 12C, the total tube length, number of nodes, number of junctions, number of master segments, total master segments length, number of meshs, total mesh area, number of branches and total branch length were 65.7% (*P* < 0.0001), 42.9% (*P* < 0.0001), 43.3% (*P* < 0.0001), 33.3% (*P* < 0.0001), 49.5% (*P* < 0.0001), 24.3% (*P* < 0.0001), 32.4% (*P* < 0.0001), 105.7% (*P* = 0.5574) and 125.2% (*P* = 0.0023) relative to the mean value measured in the control cybrid cell lines respectively.

## DISCUSSION

The tissue specificity of pathogenic mitochondrial tRNA mutations remains largely elusive. The pathogenic mechanism behind the tissue-specific manifestations are likely to involve cell type-specific mitochondrial functions (65). Using HUVEC-derived cybrids under constant nuclear and mitochondrial genetic backgrounds, in combination with functional assays, we demonstrated the deleterious effects of tRNA^Thr^ 15927G>A mutation that contributed to pathogenesis of coronary artery disease, including mitochondrial dysfunctions, increasing mitochondrial ROS production, promoting apoptosis and altered angiogenesis. In fact, the m.15927G>A mutation abolished the highly conserved base-pairing (28C-42G) of anticodon stem of tRNA^Thr^ along with the 29A**·**41C mismatch (28–30). All the nucleotides in the anticodon stem and loop are critical for a proper helical conformation during genetic code decoding (66). Therefore, we hypothesized that the m.15927G>A mutation altered both structure and function of tRNA^Thr^. MD simulation results indicated that the mutant tRNA^Thr^ structure harboring the m.15927G>A mutation was more unstable than that of wild-type counterpart. Furthermore, the m.15927G>A mutation changed the conformation of tRNA^Thr^, as suggested by slower electrophoretic mobility of mutated tRNA with respect to the wild-type molecule, in contrast with the faster electrophoretic mobility of mutated tRNAs carrying the m.4435A>G and m.3253T>C mutations (22,24). In addition, the melting temperature in mutant tRNA^Thr^ (A42), decreased ∼2°C, as compared with wild type counterpart (G42). These data indicated that the m.15927G>A mutation ablated the tertiary structure and stability of tRNA^Thr^. Moreover, the G42 is a nucleotide determinant for the t^6^A modification at A37 of tRNA (57,66). Here, the deficient t^6^A37 modification of mutant tRNA^Thr^ observed *in vitro* assays was consistent with the previous investigation (57). The altered tertiary structure and deficient t^6^A37 modification of tRNA^Thr^ contributed to the decrease in the steady-state level of tRNA^Thr^. In the present study, 25% reduction in the steady-state level of tRNA^Thr^ observed in mutant cybrids was in contrast with 80% reductions in the steady state level of tRNA^Thr^ observed in the mutant lymphoblastoid cell lines (27). Alternatively, the altered tertiary structure caused by the m.15927G>A mutation may perturb the aminoacylation of tRNA^Thr^ by either charging inefficiently or mischarging with mitochondrial threonyl-tRNA synthetase (28,29). In the present study, 48% decrease aminoacylation of mutant tRNA^Thr^ transcript *in vitro* assays and 32% reduction in the efficiencies of aminoacylated tRNA^Thr^ in mutant cybrids were consistent with 39% reduction in those from lymphoblastoid cell lines (27). However, the aminoacylation levels of tRNAs were much higher in HUVEC derived cybrid cell lines than those in lymphoblastoid cell lines. As shown in Table 1, the aminoacylated levels of tRNA^Thr^, tRNA^Ile^, tRNA^Leu(CUN)^ and tRNA^Ser(AGY)^ in the control HUVEC-derived cybrids were 151%, 118%, 148% and 169% of the average values in control lymphoblastoid cell lines, respectively. These discrepancies may be attributed to the tissue specific effects in mitochondrial tRNA metabolism (67–70).

**Table 1.** Aminoacylation levels of mt-tRNAs in HUVEC derived cybrid cell lines and lymphoblastoid cell lines carrying the m.15927 G>A mutation

	Average value ± SD in HUVECs and HUVEC derived cybrids (%)			Average value ± SD in lymphoblastoid cells (%)^a,b^	
tRNAs	HUVECs	Control cell lines	15927 G>A mutant cell lines	Control cell lines	15927 G>A mutant cell lines
tRNA^Thr^	65.28 ± 3.01	73.73 ± 6.15	50.34 ± 3.60	48.83 ± 0.63	31.16 ± 5.01
tRNA^Ile^	52.18 ± 0.96	52.94 ± 1.70	51.86 ± 3.66	44.93 ± 1.78	N
tRNA^Leu(CUN)^	39.21 ± 0.23	36.62 ± 3.02	36.23 ±2.11	25.18 ±1.49	22.27 ± 2.97
tRNA^Ser(AGY)^	54.95 ± 1.28	52.89 ± 5.78	54.00 ± 4.12	31.33 ± 1.65	31.79 ± 1.89

N, no relevant data.

^a^Jia *et al.* (27).

^b^Wang *et al.* (19).

The aberrant tRNA metabolism, including inefficient aminoacylation and shortage of tRNA^Thr^ led to the impairment of mitochondrial translation and respiration. Alternatively, the mutant tRNA^Thr^ with deficient t^6^A37 modification may not interact correctly with the translational machinery, thereby altering mitochondrial protein synthesis. In fact, the mtDNA encoded 13 polypeptides in the complexes of the OXPHOS system (ND1-6, ND4L of complex I; CYTB of complex III; CO1, CO2 and CO3 of complex IV; ATP6 and ATP8 of complex V) (12). In this study, we demonstrated that the m.15927G>A mutation caused 27% reduction in the overall levels of mitochondrial proteins in mutant cybrids. These results were in contrast with 53% reductions in the rates of mitochondrial translation in lymphoblastoid cell lines carrying the m.15927G>A mutation (27). However, the variable decreases in the levels of ND1, ND3, ND4, ND5, CO2, CYTB, ATP6 and ATP8 were observed in the mutant cell lines, whereas there were no difference the levels of ND6 with lower threonine codons between mutant and control cell lines (Supplemental Table 2). In contrast to what was previously shown in cells carrying the tRNA^Lys^ 8344A>G and tRNA^Ser(UCN)^ 7445A>G mutations, the reduced levels of these polypeptides in mutant cybrid cell lines did not significantly correlate with the number or proportion of threonine codons (71,72). The impairment of mitochondrial translation led to reduced rates in the basal OCR, ATP-linked OCR, maximal OCR and reserve capacity in the mutant cybrid cell lines. These results highlighted that aberrant tRNA metabolism played a critical role in producing their respiratory deficiency, as in the cases of cells carrying hypertension-associated tRNA^Ala^ 5655A>G and tRNA^Leu(UUR)^ 3253T>C mutations (23,24).

The respiratory deficiency caused by the m.15927G>A mutation resulted in the alterations on ATP synthesis, mitochondrial membrane potentials, oxidative stress and subsequent failure of cellular energetic process, as in the case of other mtDNA mutations (23,24,35,73). In this investigation, 49% reductions in mitochondrial membrane potential observed in mutant cybrids indicated the impaired pumping ability of hydrogen ions across the inner membrane and more electron leakage during the process of electron transport and oxidative phosphorylation (23,36,74). Alterations in both oxidative phosphorylation and mitochondrial membrane potential elevated the mitochondrial ROS production and the subsequent bioenergetic failure in mutant cybrids. In particular, the overproduction of mitochondrial ROS plays a key role in the pathogenesis of coronary artery disease (10,75). As a result, the increased production of ROS by the m.15927G>A mutation may produce the damage of proteins, lipids and nuclear acids (76–79). Both altered mitochondrial membrane potential and overproduction of mitochondrial ROS production may promote the apoptosis (10,35). In the present investigation, mutant cybrids exhibited more apoptotic susceptibility than control cybrids lacking the mutation, evidenced by the elevated release of cytochrome *c* into cytosol and increased levels of apoptosis-activated proteins: caspases 9, 3, 7 and PARP, as compared to control cybrids. These data demonstrated that mitochondrial dysfunction caused by m.15927G>A mutations promoted the apoptosis.

Mitochondrial dysfunction plays an important role in cardiovascular pathophysiology. In particular, mitochondrial ROS production, metabolic regulation and apoptosis are essential for defining the proper balance necessary for ensure the endothelial switch towards a proliferative phenotype required for angiogenesis (80). In particular, excessive ROS production ensuring mitochondrial dysfunction in blood is responsible for inflammatory vascular reactions leading to cardiovascular diseases (77,78). Using wound healing and the tube formation assays, we demonstrated the specific effects of m.15927G>A mutation on wound regeneration and angiogenesis in HUVECs-derived cybrids, as the lower wound healing rate and perturbed tube formation. These data strongly suggested that the m.15927 G>A mutation-induced mitochondrial dysfunction lead to coronary artery disease.

In summary, our findings demonstrated the pathogenic mechanism leading to an impaired oxidative phosphorylation in HUVECs-derived cybrid cell lines carrying the CAD-associated tRNA^Thr^ 15927G>A mutation. The m.15927G>A mutation alters both structure and function of tRNA. The aberrant tRNA^Thr^ metabolism impaired mitochondrial translation, caused respiratory deficiency, diminished membrane potential and increased the production of reactive oxygen species. All those alterations consequently elevated the apoptotic cell death and altered the angiogenesis, thereby leading to coronary artery disease. Therefore, our findings provide new insights into the pathophysiology of coronary artery disease, which was manifested by mitochondrial tRNA^Thr^ mutation-induced alterations.

## Supplementary Material

Supplementary Data

## References

[1] Lopez A.D., Mathers C.D., Ezzati M., Jamison D.T., Murray C.J. Global and regional burden of disease and risk factors, 2001: systematic analysis of population health data. Lancet. 2006; 367:1747–1757. 16731270 10.1016/S0140-6736(06)68770-9

[2] Mathers C.D., Loncar D. Projections of global mortality and burden of disease from 2002 to 2030. PLoS Med. 2006; 3:e442. 17132052 10.1371/journal.pmed.0030442 PMC1664601

[3] North B.J., Sinclair D.A. The intersection between aging and cardiovascular disease. Circ. Res. 2012; 110:1097–1108. 22499900 10.1161/CIRCRESAHA.111.246876 PMC3366686

[4] Falk E. Pathogenesis of atherosclerosis. J. Am. Coll. Cardiol. 2006; 47:7–12. 10.1016/j.jacc.2005.09.068 16631513

[5] Sing C.F., Stengard J.H., Kardia S.L. Genes, environment, and cardiovascular disease. Arterioscler Thromb. Vasc. Biol. 2003; 23:1190–1196. 12730090 10.1161/01.ATV.0000075081.51227.86

[6] Pjanic M., Miller C.L., Wirka R., Kim J.B., DiRenzo D.M., Quertermous T. Genetics and genomics of coronary artery disease. Curr Cardiol Rep. 2016; 18:102. 27586139 10.1007/s11886-016-0777-y PMC5872834

[7] Dominic E.A., Ramezani A., Anker S.D., Verma M., Mehta N., Rao M. Mitochondrial cytopathies and cardiovascular disease. Heart. 2014; 100:611–618. 24449718 10.1136/heartjnl-2013-304657

[8] Ballinger S.W. Mitochondrial dysfunction in cardiovascular disease. Free Radic. Biol. Med. 2005; 38:1278–1295. 15855047 10.1016/j.freeradbiomed.2005.02.014

[9] Erin A., Lerman A., Lerman L.O. Mitochondria: a pathogenic paradigm in hypertensive renal disease. Hypertension. 2015; 65:264–270. 25403611 10.1161/HYPERTENSIONAHA.114.04598 PMC4289015

[10] Dromparis P., Michelakis E.D. Mitochondria in vascular health and disease. Annu. Rev. Physiol. 2013; 75:95–126. 23157555 10.1146/annurev-physiol-030212-183804

[11] Ryan M.T., Hoogenraad N.J. Mitochondrial-nuclear communications. Annu. Rev. Biochem. 2007; 76:701–722. 17227225 10.1146/annurev.biochem.76.052305.091720

[12] Andrews R.M., Kubacka I., Chinnery P.F., Lightowlers R.N., Turnbull D.M., Howell N. Reanalysis and revision of the Cambridge reference sequence for human mitochondrial DNA. Nat. Genet. 1999; 23:147. 10508508 10.1038/13779

[13] Griffiths E.J. Mitochondria and heart disease. Adv. Exp. Med. Biol. 2012; 942:249–267. 22399426 10.1007/978-94-007-2869-1_11

[14] Suzuki T., Nagao A., Suzuki T. Human mitochondrial tRNAs: biogenesis, function, structural aspects, and diseases. Annu. Rev. Genet. 2011; 45:299–329. 21910628 10.1146/annurev-genet-110410-132531

[15] Xue L., Wang M., Li H., Wang H., Jiang F., Hou L., Geng J., Lin Z., Peng Y., Zhou H. Mitochondrial tRNA mutations in 2070 Chinese Han subjects with hypertension. Mitochondrion. 2016; 30:208–221. 27544295 10.1016/j.mito.2016.08.008

[16] Marian A.J. Mitochondrial genetics and human systemic hypertension. Circ. Res. 2011; 108:784–786. 21454791 10.1161/CIRCRESAHA.111.242768

[17] Qin Y., Xue L., Jiang P., Xu M., He Y., Shi S., Huang Y., He J., Mo J.Q., Guan M.X. Mitochondrial tRNA variants in Chinese subjects with coronary heart disease. J. Am. Heart Assoc. 2014; 3:e000437. 24470521 10.1161/JAHA.113.000437 PMC3959674

[18] Wilson F.H., Hariri A., Farhi A., Zhao H., Petersen K.F., Toka H.R., Nelson-Williams C., Raja K.M., Kashgarian M., Shulman G.I. A cluster of metabolic defects caused by mutation in a mitochondrial tRNA. Science. 2004; 306:1190–1194. 15498972 10.1126/science.1102521 PMC3033655

[19] Wang S., Li R., Fettermann A., Li Z., Qian Y., Liu Y., Wang X., Zhou A., Mo J.Q., Yang L. Maternally inherited essential hypertension is associated with the novel 4263A>G mutation in the mitochondrial tRNA^Ile^ gene in a large Han Chinese family. Circ. Res. 2011; 108:862–870. 21454794 10.1161/CIRCRESAHA.110.231811

[20] Liu Y., Li R., Li Z., Wang X.J., Yang L., Wang S., Guan M.X. Mitochondrial transfer RNA^Met^ 4435A>G mutation is associated with maternally inherited hypertension in a Chinese pedigree. Hypertension. 2009; 53:1083–1090. 19398658 10.1161/HYPERTENSIONAHA.109.128702 PMC2907152

[21] Lu Z., Chen H., Meng Y., Wang Y., Xue L., Zhi S., Qiu Q., Yang L., Mo J.Q., Guan M.X. The tRNA^Met^ 4435A>G mutation in the mitochondrial haplogroup G2a1 is responsible for maternally inherited hypertension in a Chinese pedigree. Eur. J. Hum. Genet. 2011; 19:1181–1186. 21694735 10.1038/ejhg.2011.111 PMC3198143

[22] Zhou M., Xue L., Chen Y., Li H., He Q., Wang B., Meng F., Wang M., Guan M.X. A hypertension-associated mitochondrial DNA mutation introduces an m^1^G37 modification into tRNA^Met^, altering its structure and function. J. Biol. Chem. 2018; 293:1425–1438. 29222331 10.1074/jbc.RA117.000317 PMC5787817

[23] Jiang P., Wang M., Xue L., Xiao Y., Yu J., Wang H., Yao J., Liu H., Peng Y., Liu H. A hypertension-associated tRNA^Ala^ mutation alters tRNA metabolism and mitochondrial function. Mol. Cell. Biol. 2016; 36:1920–1930. 27161322 10.1128/MCB.00199-16 PMC4936059

[24] Zhou M., Wang M., Xue L., Lin Z., He Q., Shi W., Chen Y., Jin X., Li H., Jiang P. A hypertension-associated mitochondrial DNA mutation alters the tertiary interaction and function of tRNA^Leu(UUR)^. J. Biol. Chem. 2017; 292:13934–13946. 28679533 10.1074/jbc.M117.787028 PMC5572913

[25] Li R., Liu Y., Li Z., Yang L., Wang S., Guan M.X. Failures in mitochondrial tRNA^Met^ and tRNA^Gln^ metabolism caused by the novel 4401A>G mutation are involved in essential hypertension in a Han Chinese Family. Hypertension. 2009; 54:329–337. 19546379 10.1161/HYPERTENSIONAHA.109.129270 PMC2907155

[26] Li Z., Liu Y., Yang L., Wang S., Guan M.X. Maternally inherited hypertension is associated with the mitochondrial tRNAIle A4295G mutation in a Chinese family. Biochem. Biophys. Res. Commun. 2008; 367:906–911. 18177739 10.1016/j.bbrc.2007.12.150

[27] Jia Z., Wang X., Qin Y., Xue L., Jiang P., Meng Y., Shi S., Wang Y., Mo J.Q., Guan M.X. Coronary heart disease is associated with a mutation in mitochondrial tRNA. Hum. Mol. Genet. 2013; 22:4064–4073. 23736300 10.1093/hmg/ddt256 PMC3781636

[28] Florentz C., Sohm B., Tryoen-Toth P., Putz J., Sissler M. Human mitochondrial tRNAs in health and disease. Cell. Mol. Life Sci. 2003; 60:1356–1375. 12943225 10.1007/s00018-003-2343-1 PMC11138538

[29] Giege R., Sissler M., Florentz C. Universal rules and idiosyncratic features in tRNA identity. Nucleic Acids Res. 1998; 26:5017–5035. 9801296 10.1093/nar/26.22.5017 PMC147952

[30] Wang X., Lu J., Zhu Y., Yang A., Yang L., Li R., Chen B., Qian Y., Tang X., Wang J. Mitochondrial tRNA^Thr^ G15927A mutation may modulate the phenotypic manifestation of ototoxic 12S rRNA A1555G mutation in four Chinese families. Pharmacogenet. Genomics. 2008; 18:1059–1070. 18820594 10.1097/FPC.0b013e3283131661 PMC2905378

[31] Davidson S.M, Duchen M.R. Endothelial mitochondria: contributing to vascular function and disease. Circ. Res. 2007; 100:1128–1141. 17463328 10.1161/01.RES.0000261970.18328.1d

[32] Park H.J., Zhang Y., Georgescu S.P., Johnson K.L., Kong D., Galper J.B. Human umbilical vein endothelial cells and human dermal microvascular endothelial cells offer new insights into the relationship between lipid metabolism and angiogenesis. Stem Cell Rev. 2006; 2:93–102. 17237547 10.1007/s12015-006-0015-x

[33] Trounce I., Wallace D.C. Production of transmitochondrial mouse cell lines by cybrid rescue of rhodamine-6G pre-treated L-cells. Somat. Cell. Mol. Genet. 1996; 22:81–85. 8643997 10.1007/BF02374379

[34] Williams A.J., Murrell M., Brammah S., Minchenko J., Christodoulou J. A novel system for assigning the mode of inheritance in mitochondrial disorders using cybrids and rhodamine 6G. Hum. Mol. Genet. 1999; 8:1691–1697. 10441332 10.1093/hmg/8.9.1691

[35] Zhang J., Ji Y., Lu Y., Fu R., Xu M., Liu X., Guan M.X. Leber's hereditary optic neuropathy (LHON)-associated ND5 12338T > C mutation altered the assembly and function of complex I, apoptosis and mitophagy. Hum. Mol. Genet. 2018; 27:1999–2011. 29579248 10.1093/hmg/ddy107

[36] Rieder M.J., Taylor S.L., Tobe V.O., Nickerson D.A. Automating the identification of DNA variations using quality-based fluorescence re-sequencing: analysis of the human mitochondrial genome. Nucleic Acids Res. 1998; 26:967–973. 9461455 10.1093/nar/26.4.967 PMC147367

[37] Pettersen E.F., Goddard T.D., Huang C.C., Couch G.S., Greenblatt D.M., Meng E.C., Ferrin T.E. UCSF Chimera–a visualization system for exploratory research and analysis. J. Comput. Chem. 2004; 25:1605–1612. 15264254 10.1002/jcc.20084

[38] Case D.A., Cheatham T.E., Darden T., Gohlke H., Luo R., Merz K.M., Onufriev A., Simmerling C., Wang B., Woods R.J. The Amber biomolecular simulation programs. J. Comput. Chem. 2005; 26:1668–1688. 16200636 10.1002/jcc.20290 PMC1989667

[39] Ryckaert J.P., Ciccotti G., Berendsen H.J.C. Numerical integration of the Cartesian equations of motion of a system with constraints: Molecular dynamics of n-alkanes. J. Comput. Phys. 1977; 23:327–341.

[40] Wang M., Liu H., Zheng J., Chen B., Zhou M., Fan W., Wang H., Liang X., Zhou X., Eriani G. A Deafness- and Diabetes-associated tRNA Mutation Causes Deficient Pseudouridinylation at Position 55 in tRNA^Glu^ and Mitochondrial Dysfunction. J. Biol. Chem. 2016; 291:21029–21041. 27519417 10.1074/jbc.M116.739482 PMC5076513

[41] Li Y., Chen J., Wang E., Wang Y. T7 RNA polymerase transcription of *Escherichia coli* isoacceptors tRNA^Leu^. Sci. China C. Life Sci. 1999; 42:185–190. 18726472 10.1007/BF02880055

[42] Wang Y., Zeng Q.Y., Zheng W.Q., Ji Q.Q., Zhou X.L., Wang E.D. A natural non-Watson–Crick base pair in human mitochondrial tRNA^Thr^ causes structural and functional susceptibility to local mutations. Nucleic Acids Res. 2018; 46:4662–4676. 29648639 10.1093/nar/gky243 PMC5961198

[43] King M.P., Attardi G. Post-transcriptional regulation of the steady-state levels of mitochondrial tRNAs in HeLa cells. J. Biol. Chem. 1993; 268:10228–10237. 7683672

[44] Gong S., Peng Y., Jiang P., Wang M., Fan M., Wang X., Zhou H., Li H., Yan Q., Huang T. A deafness-associated tRNA^His^ mutation alters the mitochondrial function, ROS production and membrane potential. Nucleic Acids Res. 2014; 42:8039–8048. 24920829 10.1093/nar/gku466 PMC4081083

[45] Wang M., Peng Y., Zheng J., Zheng B., Jin X., Liu H., Wang Y., Tang X., Huang T., Jiang P. A deafness-associated tRNA^Asp^ mutation alters the m1G37 modification, aminoacylation and stability of tRNA^Asp^ and mitochondrial function. Nucleic Acids Res. 2016; 44:10974–10985. 27536005 10.1093/nar/gkw726 PMC5159531

[46] Jiang P., Jin X., Peng Y., Wang M., Liu H., Liu X., Zhang Z., Ji Y., Zhang J., Liang M. The exome sequencing identified the mutation in YARS2 encoding the mitochondrial tyrosyl-tRNA synthetase as a nuclear modifier for the phenotypic manifestation of Leber's hereditary optic neuropathy-associated mitochondrial DNA mutation. Hum. Mol. Genet. 2016; 25:584–596. 26647310 10.1093/hmg/ddv498

[47] Enriquez J.A., Attardi G. Analysis of aminoacylation of human mitochondrial tRNAs. Methods Enzymol. 1996; 264:183–196. 8965692 10.1016/s0076-6879(96)64019-1

[48] Dranka B.P., Benavides G.A., Diers A.R., Giordano S., Zelickson B.R., Reily C., Zou L., Chatham J.C., Hill B.G., Zhang J. Assessing bioenergetic function in response to oxidative stress by metabolic profiling. Free Radic. Biol. Med. 2011; 51:1621–1635. 21872656 10.1016/j.freeradbiomed.2011.08.005 PMC3548422

[49] Reers M., Smiley S.T., Mottola-Hartshorn C., Chen A., Lin M., Chen L.B. Mitochondrial membrane potential monitored by JC-1 dye. Methods Enzymol. 1995; 260:406–417. 8592463 10.1016/0076-6879(95)60154-6

[50] Mahfouz R., Sharma R., Lackner J., Aziz N., Agarwal A. Evaluation of chemiluminescence and flow cytometry as tools in assessing production of hydrogen peroxide and superoxide anion in human spermatozoa. Fertil. Steril. 2009; 92:819–827. 18710706 10.1016/j.fertnstert.2008.05.087

[51] Meng F., He Z., Tang X., Zheng J., Jin X., Zhu Y., Ren X., Zhou M., Wang M., Gong S. Contribution of the tRNA^Ile^ 4317A→G mutation to the phenotypic manifestation of the deafness-associated mitochondrial 12S rRNA 1555A→G mutation. J. Biol. Chem. 2018; 293:3321–3334. 29348176 10.1074/jbc.RA117.000530 PMC5836119

[52] Zhang J., Liu X., Liang X., Lu Y., Zhu L., Fu R., Ji Y., Fan W., Chen J., Lin B. A novel ADOA-associated OPA1 mutation alters the mitochondrial function, membrane potential, ROS production and apoptosis. Sci. Rep. 2017; 7:5704. 28720802 10.1038/s41598-017-05571-y PMC5515948

[53] Jonkman J.E., Cathcart J.A., Xu F., Bartolini M.E., Amon J.E., Stevens K.M., Colarusso P. An introduction to the wound healing assay using live-cell microscopy. Cell Adh. Migr. 2014; 8:440–451. 25482647 10.4161/cam.36224 PMC5154238

[54] DeCicco-Skinner K.L., Henry G.H., Cataisson C., Tabib T., Gwilliam J.C., Watson N.J., Bullwinkle E.M., Falkenburg L., O’Neill R.C., Morin A. Endothelial cell tube formation assay for the in vitro study of angiogenesis. J. Vis. Exp. 2014; 91:e51312. 10.3791/51312 PMC4540586 25225985

[55] Francescone RA 3rd, Faibish M, Shao R A Matrigel-based tube formation assay to assess the vasculogenic activity of tumor cells. J. Vis. Exp. 2011; 55:e3040. 10.3791/3040 PMC3230200 21931289

[56] Meng F., Cang X., Peng Y., Li R., Zhang Z., Li F., Fan Q., Guan A.S., Fischel-Ghosian N., Zhao X. Biochemical evidence for a nuclear modifier allele (A10S) in TRMU (Methylaminomethyl-2-thiouridylate-methyltransferase) related to mitochondrial tRNA modification in the phenotypic manifestation of deafness-associated 12S rRNA mutation. J. Biol. Chem. 2017; 292:2881–2892. 28049726 10.1074/jbc.M116.749374 PMC5314183

[57] Lin H., Miyauchi K., Harada T., Okita R., Takeshita E., Komaki H., Fujioka K., Yagasaki H., Goto Y.I., Yanaka K. CO2-sensitive tRNA modification associated with human mitochondrial disease. Nat. Commun. 2018; 9:1875. 29760464 10.1038/s41467-018-04250-4 PMC5951830

[58] Ojala D., Montoya J., Attardi G. tRNA punctuation model of RNA processing in human mitochondria. Nature. 1981; 290:470–474. 7219536 10.1038/290470a0

[59] Mercer T.R., Neph S., Dinger M.E., Crawford J., Smith M.A., Shearwood A.M., Haugen E., Bracken C.P., Rackham O., Stamatoyannopoulo s J.A. The human mitochondrial transcriptome. Cell. 2011; 146:645–658. 21854988 10.1016/j.cell.2011.06.051 PMC3160626

[60] Scheffler I.E. Mitochondrial disease associated with complex I (NADH-CoQ oxidoreductase) deficiency. J. Inherit. Metab. Dis. 2015; 38:405–415. 25224827 10.1007/s10545-014-9768-6

[61] Sena L.A., Chandel N.S. Physiological roles of mitochondrial reactive oxygen species. Mol. Cell. 2012; 48:158–167. 23102266 10.1016/j.molcel.2012.09.025 PMC3484374

[62] Jiang X., Wang X. Cytochrome C-mediated apoptosis. Annu. Rev. Biochem. 2004; 73:87–106. 15189137 10.1146/annurev.biochem.73.011303.073706

[63] Taylor R. C., Cullen S. P., Martin S. J. Apoptosis: controlled demolition at the cellular level. Nat. Rev. Mol. Cell Biol. 2008; 9:231–241. 18073771 10.1038/nrm2312

[64] Guo S., DiPietro L.A. Factors affecting wound healing. J. Dent. Res. 2010; 89:219–229. 20139336 10.1177/0022034509359125 PMC2903966

[65] Nunnari J., Suomalainen A. Mitochondria: in sickness and in health. Cell. 2012; 148:1145–1159. 22424226 10.1016/j.cell.2012.02.035 PMC5381524

[66] Grosjean H., Westhof E. An integrated, structure- and energy-based view of the genetic code. Nucleic Acids Res. 2016; 44:8020–8040. 27448410 10.1093/nar/gkw608 PMC5041475

[67] Dittmar K.A., Goodenbour J.M., Pan T. Tissue-specific differences in human transfer RNA expression. PLoS Genet. 2006; 2:e221. 17194224 10.1371/journal.pgen.0020221 PMC1713254

[68] Tischner C., Hofer A., Wulff V., Stepek J., Dumitru I., Becker L., Haack T., Kremer L., Datta A.N., Sperl W. MTO1 mediates tissue specificity of OXPHOS defects via tRNA modification and translation optimization, which can be bypassed by dietary intervention. Hum. Mol. Genet. 2015; 24:2247–2266. 25552653 10.1093/hmg/ddu743 PMC4380071

[69] Zhang Q., Zhang L., Chen D., He X., Yao S., Zhang Z., Chen Y., Guan M.X. Deletion of Mtu1 (Trmu) in zebrafish revealed the essential role of tRNA modification in mitochondrial biogenesis and hearing function. Nucleic Acids Res. 2018; 46:10930–10945. 30137487 10.1093/nar/gky758 PMC6237746

[70] Dogan S.A., Pujol C., Maiti P., Kukat A., Wang S., Hermans S., Senft K., Wibom R., Rugarli E.I., Trifunovic A. Tissue-specific loss of DARS2 activates stress responses independently of respiratory chain deficiency in the heart. Cell Metab. 2014; 19:458–469. 24606902 10.1016/j.cmet.2014.02.004

[71] Enriquez J.A., Chomyn A., Attardi G. MtDNA mutation in MERRF syndrome causes defective aminoacylation of tRNA^Lys^ and premature translation termination. Nat Genet. 1995; 10:47–55. 7647790 10.1038/ng0595-47

[72] Guan M.X., Enriquez J.A., Fischel-Ghodsian N., Puranam R.S., Lin C.P., Maw M.A., Attardi G. The deafness-associated mitochondrial DNA mutation at position 7445, which affects tRNA^Ser(UCN)^ precursor processing, has long-range effects on NADH dehydrogenase subunit ND6 gene expression. Mol. Cell. Biol. 1998; 18:5868–5879. 9742104 10.1128/mcb.18.10.5868 PMC109173

[73] Wallace D.C. A mitochondrial paradigm of metabolic and degenerative diseases, aging, and cancer: a dawn for evolutionary medicine. Annu. Rev. Genet. 2005; 39:359–407. 16285865 10.1146/annurev.genet.39.110304.095751 PMC2821041

[74] Jiang P., Liang M., Zhang C., Zhao X., He Q., Cui L., Liu X., Sun Y.H., Fu Q., Ji Y. Biochemical evidence for a mitochondrial genetic modifier in the phenotypic manifestation of Leber's hereditary optic neuropathy-associated mitochondrial DNA mutation. Hum. Mol. Genet. 2016; 25:3613–3625. 27427386 10.1093/hmg/ddw199 PMC6281387

[75] Marcu R., Zheng Y., Hawkins B.J. Mitochondria and angiogenesis. Adv. Exp. Med. Biol. 2017; 982:371–406. 28551799 10.1007/978-3-319-55330-6_21

[76] Schapira A.H. Mitochondrial diseases. Lancet. 2012; 379:1825–1834. 22482939 10.1016/S0140-6736(11)61305-6

[77] Murphy M.P. Understanding and preventing mitochondrial oxidative damage. Biochem. Soc. Trans. 2016; 44:1219–1226. 27911703 10.1042/BST20160108 PMC5095902

[78] Hayashi G., Cortopassi G. Oxidative stress in inherited mitochondrial diseases. Free Radic. Biol. Med. 2015; 88A:10–17. 10.1016/j.freeradbiomed.2015.05.039 PMC4593728 26073122

[79] Schieber M., Chandel N.S. ROS function in redox signaling and oxidative stress. Curr. Biol. 2014; 24:R453–R462. 24845678 10.1016/j.cub.2014.03.034 PMC4055301

[80] Zhang D.X., Gutterman DD. Mitochondrial reactive oxygen species-mediated signaling in endothelial cells. Am. J. Physiol. Heart. Circ. Physiol. 2007; 292:H2023–H2031. 17237240 10.1152/ajpheart.01283.2006

