# Supplementary material for: A coronary artery disease-associated tRNAThr mutation altered mitochondrial function, apoptosis and angiogenesis
Source: Nucleic Acids Res. 2018 Dec 12;47(4):2056–74. doi: 10.1093/nar/gky1241 (PMC6393294; doi:10.1093/nar/gky1241)
Supplement: Supplementary Data [file gky1241_supplemental_files.pdf]

SUPPLEMENTAL DATA

Supplemental Figure S1

Supplemental Figure S2

Supplemental Figure S3

Supplemental Table S1

Supplemental Table S2

Supplemental Figure S1

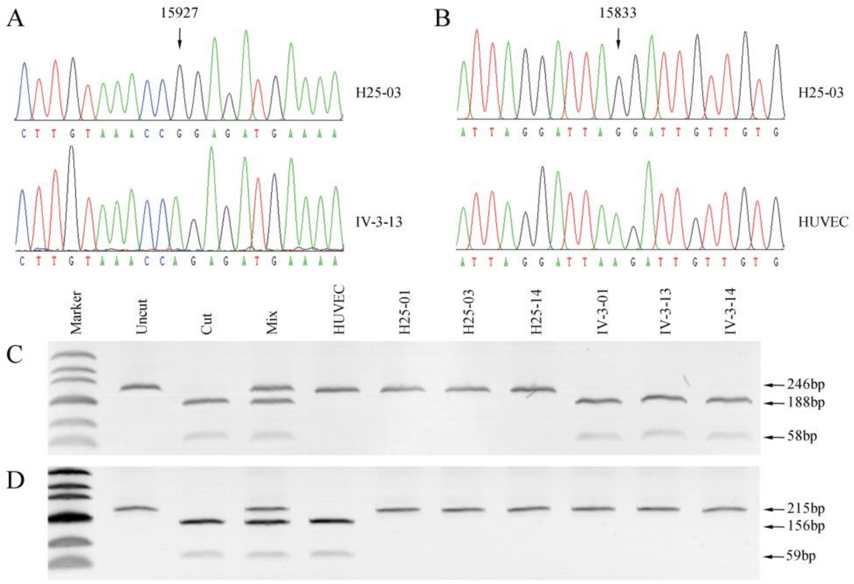

Supplemental Figure S1. Identification and quantification of the m.15927 G>A mutation and m.15833 C>T variant in the tRNA^{Thr} gene. (A) Sequence electropherograms of the mtDNA fragments covering position 15927 from the cybrid cell lines of an affected individual (IV-3) and one genetically unrelated control individual (HZC25) respectively. An arrow indicates the location of the base changes at position 15927. (B) Sequence electropherograms of the mtDNA fragments covering position 15833 from the control individual (HZC25) and HUVECs respectively. An arrow indicates the location of the base changes at position 15833. Quantification of the m.15927 G>A mutation (C) and m.15833 C>T variant (D) by PCR-RFLP. PCR products were digested with Bgl II (15927) and Hind III (15833) analyzed by electrophoresis in a 10% polyacrylamide gel stained with ethidium bromide.

Supplemental Figure S2

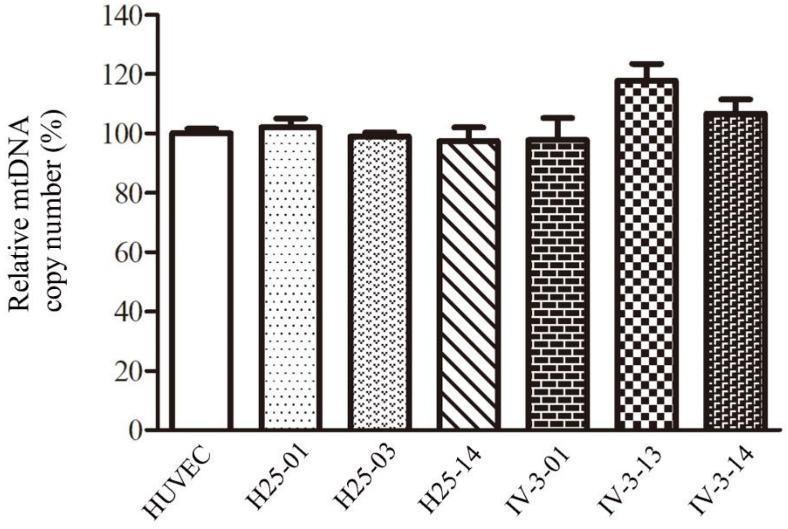

Supplemental Figure S2. Measurements of mtDNA copy numbers in cybrid cell lines. The mtDNA copy numbers were determined by comparing the ratio of mtDNA to nDNA (18S) by real-time quantitative PCR. The calculations were based on three independent determinations. The *error bars* indicate two standard errors of the means.

Supplemental Figure S3

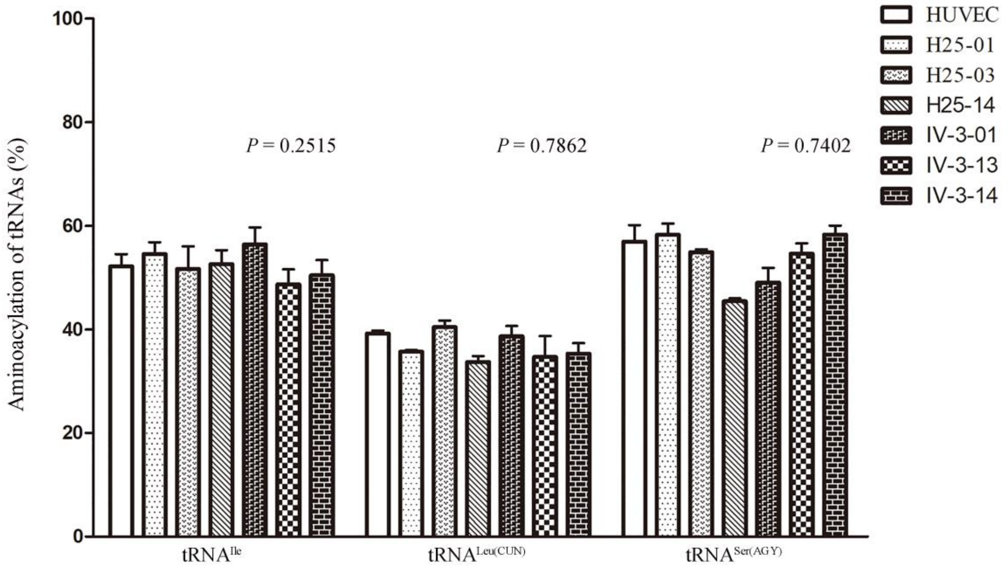

Supplemental Figure S3. Relative *in vivo* aminoacylated proportions of $tRNA^{Ile}$, $tRNA^{Leu(CUN)}$ and $tRNA^{Ser(AGY)}$ in the HUVEC, mutant and control cybrid cell lines. The calculations were based on three independent determinations. The *error bars* indicate two standard errors of the means, the horizontal dashed lines represent the average value for each group. *P* indicates the signifcance, according to the t-test, of the differences between mutant and control cybrid cell lines.

Supplemental Table S1. mtDNA variants in HUVEC, one Chinese subject (IV-4) with CHD and one control Chinese control subject (HZC25).

Gene	Position	Replacement	CRS [a]	HUVEC	HZC25	BJH45 IV-3	Previous reported [b]
D-loop	73	A to G	A		G	G	Yes
	103	G to A	G			A	Yes
	210	A to G	A		G		Yes
	227	A to T	A		T		Yes
	263	A to G	A	G	G	G	Yes
	310	T to CTC	T		CTC	CTC	Yes
	315	C to CC	C	CC			Yes
	414	T to G	T	G			Yes
	444	A to G	A	G			Yes
	456	C to T	C	T			Yes
	481	C to T	C			T	Yes
	514	C to Del	C			Del C	Yes
	515	A to Del	A			Del A	Yes
	522	C to Del	C	Del C			Yes
	523	A to Del	A	Del A			Yes
	16093	T to C	T	C			Yes
	16140	T to C/CTC	T		CTC	C	Yes
	16172	T to C	T	C			Yes
	16183	A to C	A			C	Yes
	16189	T to C	T		C	C	Yes
	16193	C to CC	C			CC	Yes
	16234	C to T	C			T	Yes
	16243	T to C	T			C	Yes
	16266	C to G	C		G		Yes
	16304	T to C	T			C	Yes
	16344	C to T	C			T	Yes
	16463	A to G	A			G	Yes
	16519	T to C	T		C	C	Yes
12S rRNA	709	G to A	G		A	A	Yes
	750	A to G	A	G	G	G	Yes
	1438	A to G	A	G	G	G	Yes
	1598	G to A	G			A	Yes
16S rRNA	2706	A to G	A		G	G	Yes
ND1	3537	A to G	A		G		Yes
	3745	G to A (Ala to Thr)	G	A			Yes
	4161	C to T	C			T	Yes
$tRNA^{Gln}$	4336	T to C	T	C			Yes
ND2	4769	A to G	A	G		G	Yes
	4895	A to G	A			G	Yes

	5178	C to G	C		G		Yes
CO1	6663	A to G (Ile to Val)	A		G		Yes
	7028	C to T	C		T	T	Yes
NC	8271-8279	9bp Del	C		9bp Del	9bp Del	Yes
ATP6	8584	G to A (Ala to Thr)	G		A	A	Yes
	8829	C to T	C			T	Yes
	8860	A to G (Thr to Ala)	A	G	G	G	Yes
	8902	G to A (Ala to Thr)	G		A		Yes
	9100	A to G (Ile to Val)	A		G		Yes
CO3	9804	G to A (Ala to Thr)	G	A			Yes
	9950	T to C	T		C	C	Yes
ND3	10398	A to G (Thr to Ala)	A		G	G	Yes
ND4	11101	A to G	A			G	Yes
	11719	G to A	G		A	A	Yes
ND5	12361	A to G (Thr to Ala)	A			G	Yes
ND6	14221	T to C	T			C	Yes
CYTB	14766	C to T (Thr to Ile)	C		T	T	Yes
	15077	G to A (Glu to Lys)	G		A		Yes
	15223	C to T	C			T	Yes
	15235	A to G	A		G		Yes
	15263	C to T (Pro to Ser)	C		T		Yes
	15326	A to G (Thr to Ala	A	G	G	G	Yes
	15508	C to T	C			T	Yes
	15662	A to G (Ile to val)	A			G	Yes
	15777	G to A (Ser to Asn)	G		A		Yes
	15833	C to T	C	T			Yes
	15850	T to C	T			C	Yes
	15851	A to G (Ile to Val)	A			G	Yes
$tRNA^{Thr}$	15927	G to A	G			A	Yes

[a] CRS: Cambridge reference sequence (12);

[b] See the online mitochondrial genome database http://www.mitomap.org and http://www.genpat.uu.se/mtDB/

Supplemental Table 2. Usage of threonine codons in human mitochondrial genes and average levels of individual polypeptide in mutant cell lines carrying the m.15927 G>A mutation, related to the average values in the control cell lines

**Proteins**	**Number of amino acids**	**Number of threonine codons**	**Density of threonine codons (%)**	**Decrease level of mitochondrial proteins (%)**
ATP8	68	8	11.8	27.42
ATP6	226	25	11.1	33.29
ND1	318	35	11.0	17.62
ND5	603	65	10.8	34.76
ND4	459	48	10.5	39.62
COX II	227	21	9.3	29.51
CYTB	380	30	7.9	27.74
ND3	115	7	6.1	29.66
ND6	174	3	1.7	- 6.43
ND2	347	43	12.4	NA
COX III	261	24	9.2	NA
COX I	513	34	6.6	NA
ND4L	98	5	5.1	NA

NA, not applicable.
